# Alcobiosis, an algal-fungal association on the threshold of lichenisation

**DOI:** 10.1038/s41598-023-29384-4

**Published:** 2023-02-28

**Authors:** Jan Vondrák, Stanislav Svoboda, Lucie Zíbarová, Lenka Štenclová, Jan Mareš, Václav Pouska, Jiří Košnar, Jiří Kubásek

**Affiliations:** 1 Institute of Botany of the Czech Academy of Sciences, Zámek 1, CZ‐252 43 Průhonice, Czech Republic; 2 Department of Botany, Faculty of Science, University of South Bohemia CZ, 370 05 České Budějovice, Czech Republic; 3 Independent Researcher, Resslova 26, 400 01 Usti Nad Labem, Czech Republic; 4 Biology Centre of the Czech Academy of Sciences, Institute of Hydrobiology, 370 05 České Budějovice, Czech Republic; 5 Faculty of Forestry and Wood Science, Czech University of Life Sciences Prague, Kamýcká 129, 165 00 Praha-Suchdol, Czech Republic; 6 Department of Experimental Plant Biology, Faculty of Science, University of South Bohemia, CZ‐370 05 České Budějovice, Czech Republic

**Keywords:** Biological techniques, Ecology, Evolution, Physiology, Plant sciences

## Abstract

Alcobiosis, the symbiosis of algae and corticioid fungi, frequently occurs on bark and wood. Algae form a layer in or below fungal basidiomata reminiscent of the photobiont layer in lichens. Identities of algal and fungal partners were confirmed by DNA barcoding. Algal activity was examined using gas exchange and chlorophyll fluorescence techniques. Carbon transfer from algae to fungi was detected as ^13^C, assimilated by algae, transferred to the fungal polyol. Nine fungal partners scattered across Agaricomycetes are associated with three algae from Trebouxiophycae: *Coccomyxa* sp. with seven fungal species on damp wood, *Desmococcus olivaceus* and *Tritostichococcus coniocybes*, both with a single species on bark and rain-sheltered wood, respectively. The fungal partner does not cause any obvious harm to the algae. Algae enclosed in fungal tissue exhibited a substantial CO_2_ uptake, but carbon transfer to fungal tissues was only detected in the *Lyomyces-Desmococcus* alcobiosis where some algal cells are tightly enclosed by hyphae in goniocyst-like structures. Unlike lichen mycobionts, fungi in alcobioses are not nutritionally dependent on the algal partner as all of them can live without algae. We consider alcobioses to be symbioses in various stages of co-evolution, but still quite different from true lichens.

## Premises

1. **Definition of symbiosis.** Symbiosis is a commonly used term in biology, but traditionally has two distinct meanings^1^. In what follows, we use it in the sense of de Bary^2^ to refer to the close and long-term coexistence of two different organisms. Symbiosis in this sense need not involve any beneficial or harmful relationship, merely close co-existence.
2. **Definition of lichen.** Lücking et al.^3^ provided a chronologically ordered list of lichen definitions. None of these is entirely satisfactory for our purposes, so here we define a lichen as follows. A lichen is an association of a fungus (mycobiont) and an alga or cyanobacterium (photobiont) with the following characteristics: (i) The mycobiont is nutritionally dependent on its photobiont. (ii) The mycobiont is not obviously harmful to its photobiont. (iii) The photobiont occurs within the mycobiont thallus. (iv) Mycobionts and photobionts usually cannot persist over a long period outside the symbiosis.

## Introduction

Mutualistic relationship between photoautotrophs and fungi arose many times in evolution, had paramount importance in the development of terrestrial life and still remains essential in the present-day ecosystems^4^. A flagship of these relationships is lichen symbiosis, a highly elaborated cooperation between fungi and green algae and/or cyanobacteria where the fungal partner is nutritionally dependent on its photoautotroph^5^. Lichen symbiosis has multiple independent origins^6^ and its complexity and the stage of lichenisation differs considerably in various examples^7^. In some cases, a single fungal species may be either lichenised or saprophytic depending on conditions^8,9^, and the same is truth for algae^10^.

Numerous fungi are apparently associated with, and nutritionally dependent on algae, but their thallus is inconspicuous, not stratified into the typical lichen thallus^6^. A list of such fungi, so called “semilichens”, has been recently provided by Vondrák et al.^11^. Apart from semilichens, other algal-fungal symbioses that do not fully meet the definition of a lichen exist^12^. A remarkable but overlooked one, is linked to corticioid fungi, traditionally defined as basidiomycetes with fruiting bodies (basidiomata) appressed to the substrate with superficial non-poroid hymenium (extended definition in^13^). These flat basidiomata (“crusts” in further text) usually cover wood or bark.

Albertini & Schweinitz^14^ described one of the wood-dwelling corticioid fungi as *Hydnum bicolor*, currently named *Resinicium bicolor*. The epithet *bicolor* reflected the contrast between the white surface of fruiting bodies and the rusty brown tips of hymenial spines (the coloration frequently observed in older basidiomata). This epithet additionally matches an even more distinctive colour contrast—the white (or partly translucent) fungal crust is regularly green beneath. The green tinge is caused by an algal layer formed directly below the white fungal coat.

A detailed investigation of various corticioid fungi in European woodlands revealed living algal cells thriving below or inside the crusts of several unrelated fungal species. We propose to name these alliances “alcobioses” (singular “**alcobiosis**”), such as **al**ga and **co**rticioid fungus in sym**biosis**. Whereas some alcobioses form unstable associations where the algal cells are few in scattered colonies, others apparently have a tight relationship where algae form a lichen-like algal layer^15^. Only a few algal taxa have previously been reported in an association with corticioid fungi, mainly unicellular members of the green-algal class Trebouxiophyceae such as *Coccomyxa glaronensis*^15^ and undetermined species of *Coccomyxa* and *Elliptochloris*^16,17^. These algae are ubiquitous in terrestrial habitats and exhibit a general tendency to enter lichen-like symbioses^18^. However, their symbiotic and free-living members are morphologically uniform, and separation of the cryptic lineages requires molecular data^19,20^ not available for alcobioses yet.

The similarity of alcobioses and crustose lichens is remarkable, but the former have received little and only superficial attention. The question of whether alcobioses have a nutritional character, as in lichens^21^, has not been addressed. Here we provide the most comprehensive morphological and taxonomical assessment so far concerning both symbiotic partners. We also demonstrate that algal cells in these consortia are alive and metabolically and photosynthetically active even when fully embedded in the fungal crust. And we also studied carbon transfer from algal polyols (ribitol and sorbitol in our cases) into fungal mannitol that would confirm the nutritional relationship of the fungus to the algae.

## Results

### Alcobioses are stratified systems with an internal algal layer

**Figure 1:**
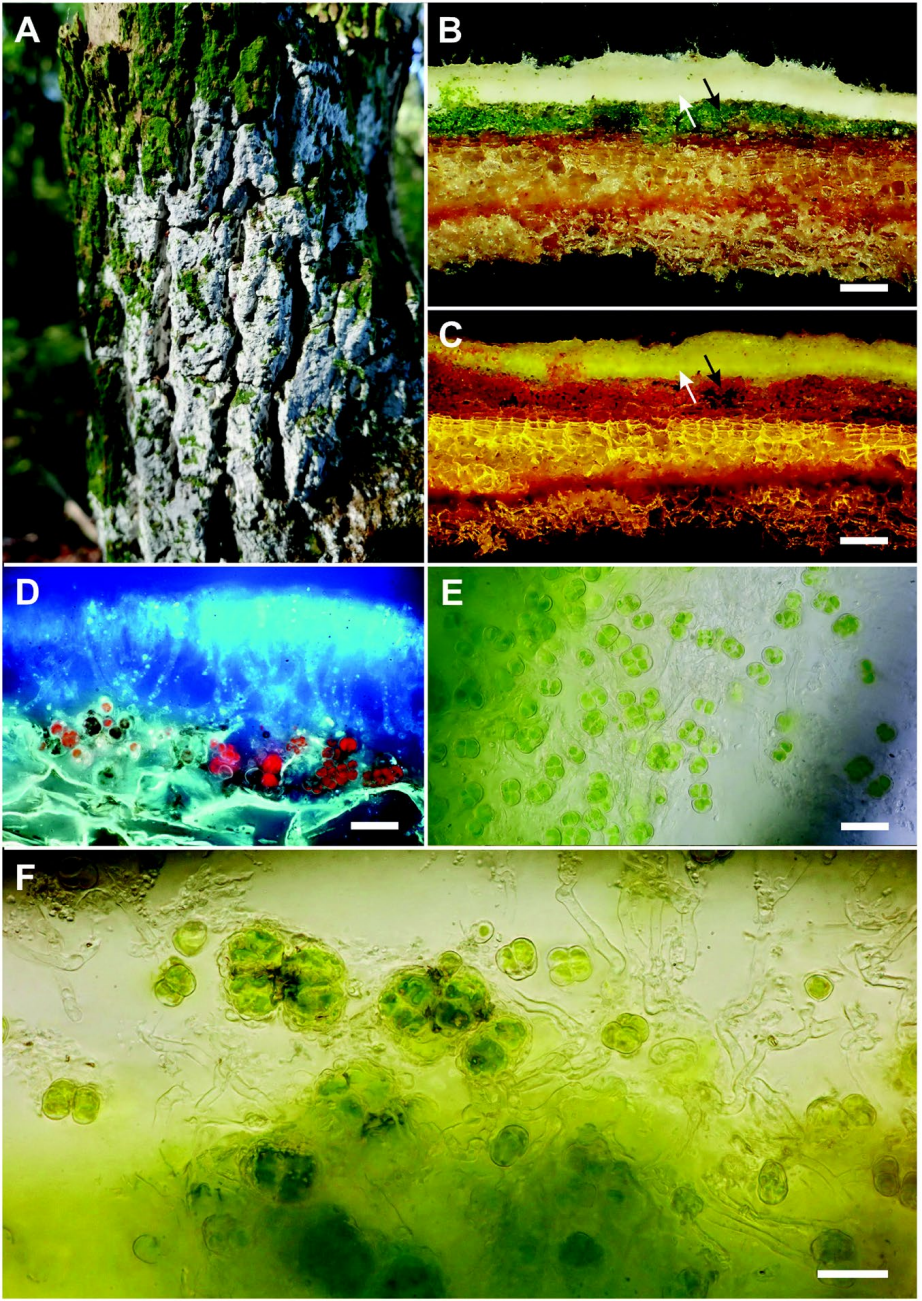
Association of *Lyomyces sambuci* and *Desmococcus olivaceus* (GPS: 48.9409975N, 14.5175219E; voucher: PRA-JV25262). (**A**) bark of *Sambucus nigra* covered by a free-living *Desmococcus* algal crust which is largely overgrown by *Lyomyces*; (**B**) vertical section of the *Lyomyces* crust with a distinct algal layer; (**C**) vertical section with the red chlorophyll autofluorescence; (**D**) algal colonies incorporated in a loose hyphal tissue, below the cover of compact fungal tissue; (**E**) *Desmococcus* in the algal layer; (**F**) *Desmococcus* and *Lyomyces* form lichen-like goniocysts. Scales: (**B**, **C**), 100 µm; (**D**, **E**, **F**), 20 µm.

**Figure 2:**
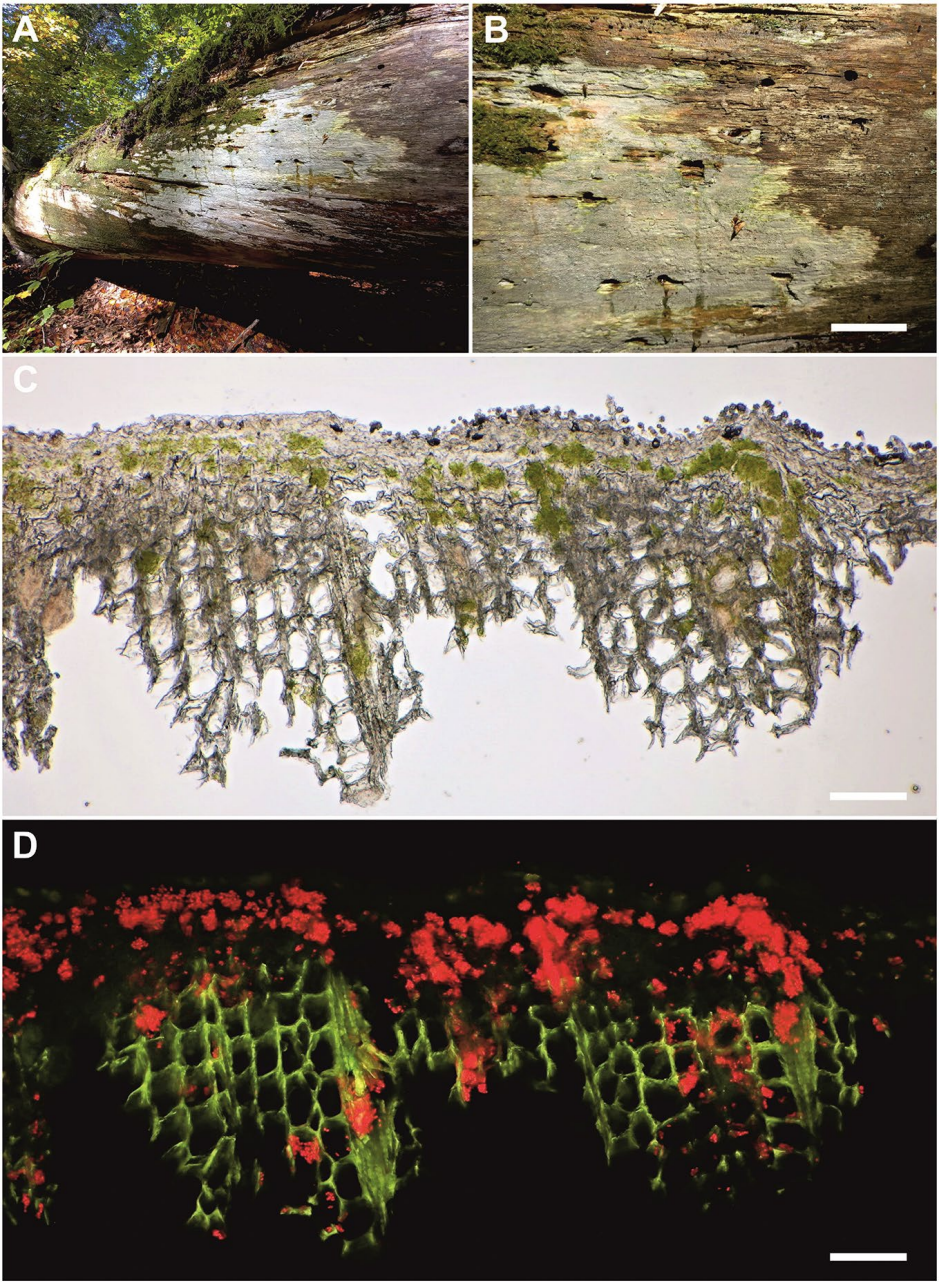
Association of *Resinicium bicolor* and *Coccomyxa* (GPS: 48.6679583N, 14.7053083E; voucher: PRA-JV25257). (**A**) typical habitat–vertical surface of rotten spruce trunks; (**B**) *R. bicolor* crust; (**C**) vertical section with a distinct algal layer below the fungal coat; (**D**) the red chlorophyll autofluorescence indicates locations of *Coccomyxa* cells in the vertical section. Scales: (**B**) 5 mm; (**C**, **D**) 50 µm.

**Figure 3:**
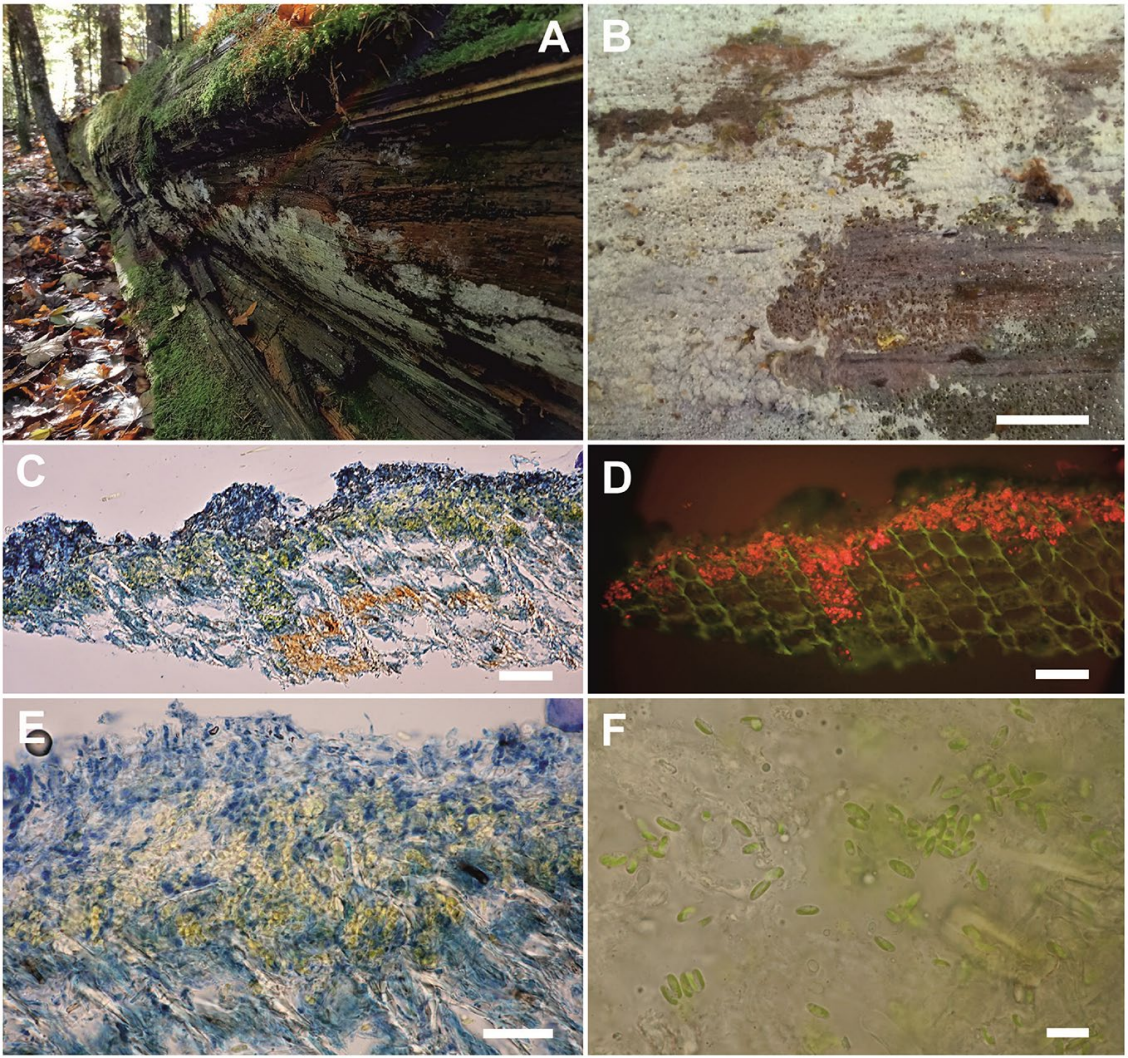
Association of *Skvortzovia furfuracea* and *Coccomyxa* (GPS: 48.6679583N, 14.7053083E; voucher: PRA-JV25255). (**A**) typical habitat – shaded surface of rotten spruce trunks; (**B**) *S. furfuracea* crust grazed by snails; (**C**, **E**) vertical sections of *S. furfuracea* crust. Fungal tissues coloured by lactoglycerol cotton blue. A distinct algal layer is visible below a dark blue fungal coat; (**D**) the red chlorophyll autofluorescence indicates locations of *Coccomyxa* cells in the vertical section; (**F**) *Coccomyxa* loosely integrated in the fungal tissue. Scales: (**B**) 1 cm; (**C**, **D**) 50 µm; (**E**) 20 µm; (**F**) 10 µm.

Algal cells were observed enclosed either in the lower part of crustose basidiomata (subiculum) or in the substratum below the crusts, mostly rotten wood, however they never covered the crust surface. The density of algal cells varied considerably among infraspecific individuals and among species from an entire absence to a distinct thick continuous layer. The thickness of the algal layer varied, but frequently exceeded 100 µm. Whereas the algal layer was formed only occasionally in some species (*Exidiopsis calcea* and *Tubulicrinis subulatus*), it was found regularly in *Lyomyces sambuci* (Fig. 1), *Resinicium bicolor* (Fig. 2), *Skvortzovia furfuracea* (Fig. 3) and some *Xylodon* spp. All these fungi, however, were also recorded living separately, without an internal algal layer. In all cases, the colonies of algal cells were enclosed in fungal tissue (although they are also found in the substrate below the crust Fig. 3E). Nevertheless, a truly close contact where algae are encircled by fungal hyphae was mostly not observed. The only exception was *Lyomyces sambuci* which occasionally formed spherical goniocyst-like structures (20–40 µm in diameter) where a group of algal cells was tightly enclosed within the hyphal network (Fig. 1F).

### Diverse fungal partners are associated with several ecologically distinct algae

**Figure 4:**
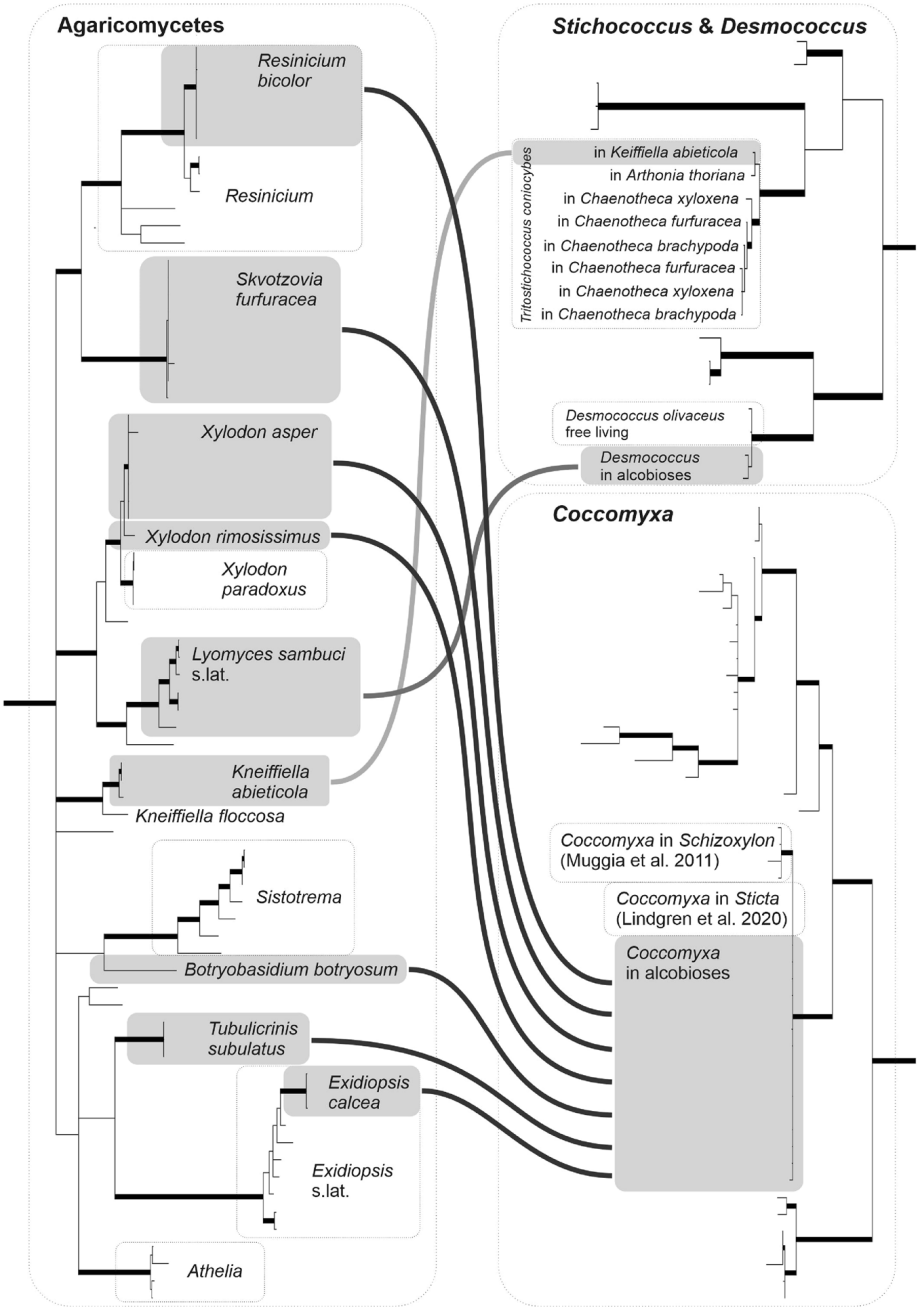
Linkage between algal and fungal partners in alcobioses. Algae are arranged in rbcL phylogenetic trees of Trebouxiophyceae; only parts with *Stichococcus* s.lat., *Desmococcus* and *Coccomyxa* depicted. Fungi are arranged in the ITS tree of selected Agaricomycetes. Symbionts in alcobioses are in grey rectangles. Detailed trees are available on Figures S3, S4, S5.

The fungal partners appear to be more diverse in alcobioses than the associated algae. We found nine fungal species dispersed across the phylogeny of Agaricomycetes (Table S1, Fig. S3) and only three algal partners from the class Trebouxiophycae (Table S1, Fig. 4, Figures S4, S5, S6). The vast majority of fungi involved (i.e. seven species) entered the symbiosis with a single *Coccomyxa* species (Figures S4, S6A,B,C,D). All these fungi have similar ecology, occurring in temperate forests on decaying wood, especially on fallen spruce trunks, in shaded conditions (Figures 2 and 3; Table S1). The associated *Coccomyxa* sp. has a *rbcL* sequence almost identical (> 99% identity) to a lichen photobiont of *Sticta*^22^ and to a symbiont in an allegedly lichenised *Schizoxylon albescens*^9^. Both symbioses have quite distinct ecology: the former is a tropical lichen from Cuba and the latter is known from aspen bark in Northern Europe. In addition, the same algal genotype labelled *Coccomyxa* sp. “cort06” in terms of the *rbcL* sequence (~99.8% identity) was found to colonize the bark of trees with no report of association with fungi^23^.

The single fungus, *Lyomyces sambuci*, was regularly associated with another green algal species *Desmococcus olivaceus*, as determined both by morphological identity (Fig. S6E,F,G,H) and high *rbcL* sequence similarity (~99.5%; Fig. S5) to a typical strain of this species—SAG 1.94^24^. This alcobiosis differs in ecology from the associations with *Coccomyxa*. It is more tolerant of drying, occurs in lighter sites and has a strong affinity to bark and wood of living or dying *Sambucus* shrubs. The alliance *Lyomyces-Desmococcus* is apparently the most intimate among the observed alcobioses as it forms goniocyst-like structures and the algal carbon is undoubtedly transferred to the fungus (see below). However, again, the same species of *Desmococcus* also occurs free-living (Fig. 1A,^24^) and its symbiosis with *Lyomyces* is clearly facultative.

Finally, a green alga matching *Tritostichococcus coniocybes,* both in morphology (Fig. S6H,I) and the *rbcL* sequence (97–99% identity; Fig. S5), was detected in alcobiosis with a single fungus, *Kneiffiella abieticola*. It was observed on soft rotten wood of spruce snag in a microsite sheltered from rain. *Tritostichococcus coniocybes* is a lichen photobiont detected in *Chaenothecopsis* spp.^24^, *Arthonia thoriana* and *Chaenotheca* spp. (Fig. 4), and also was observed free-living (our data). It always occurred in rain-sheltered microhabitats.

### Algae thrive in the association

**Figure 5:**
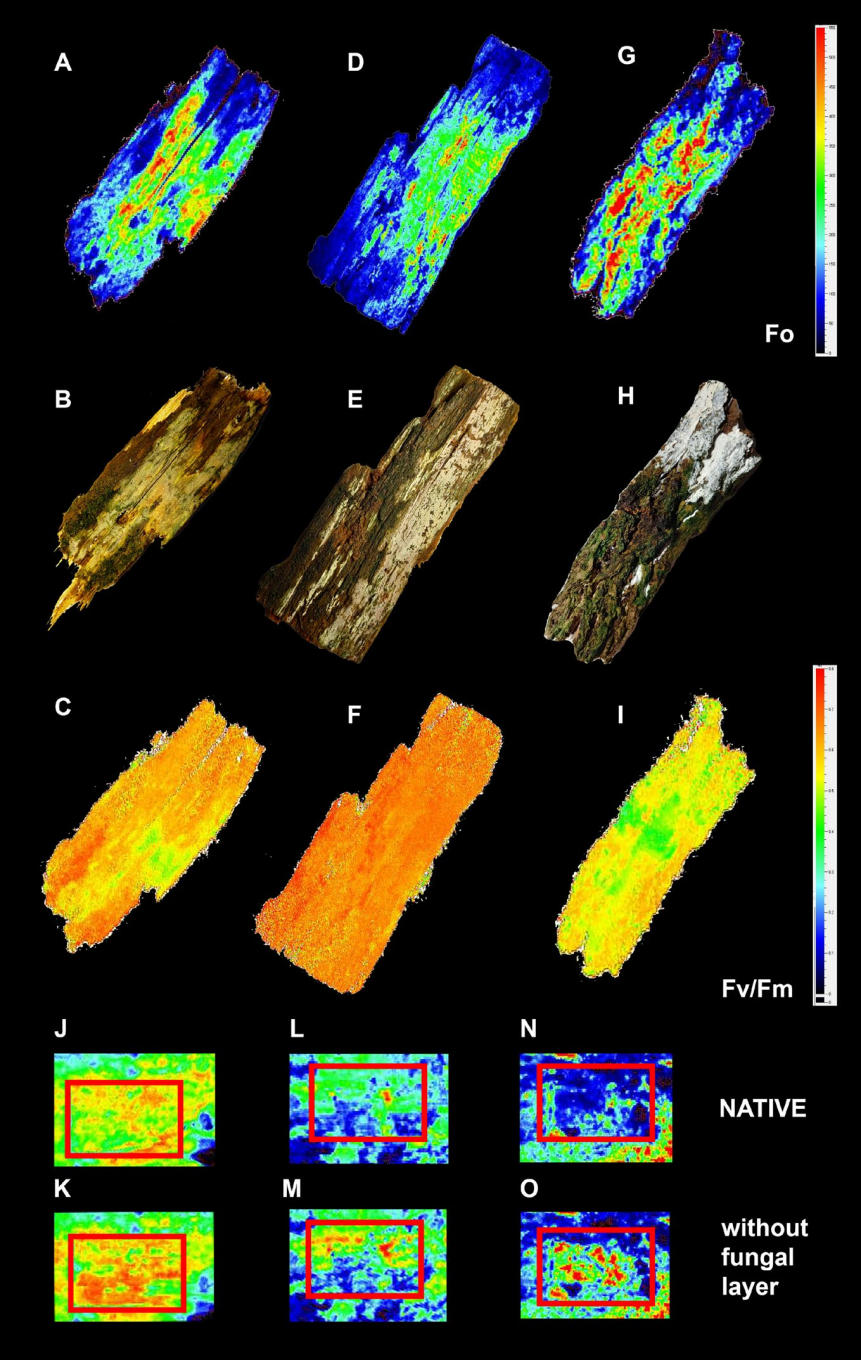
Chlorophyll fluorescence imaging of fungal-algal associations. Minimal fluorescence (F_o_), visual frame and maximal quantum yield of photosyntem II (F_v_/F_m_) for *Resinicium bicolor-Coccomyxa* (**A**, **B**, **C**, **J**, **K**), *Skvortzovia furfuracea-Coccomyxa* (**D**, **E**, **F**, **L**, **M**) and *Lyomyces sambuci-Desmococcus* (**G**, **H**, **I**, **N**, **O**). Each sample contains both algal patches and alcobiosis where fungal crust completely covers the algae. Bottom pairs of frames demonstrate shielding effect of fungal crusts. Upper frames are intact alcobioses (**J**, **L**, **N**), whereas fungal part of the system was carefully removed by razor blade in red frames of the bottoms (**K**, **M**, **O**).

Absolute fluorescence intensity (e.g. F_o_) is very variable and demonstrates heterogeneity in algal chlorophyll abundance (Fig. 5A,D,G). The maximal quantum yield of photosystem II (F_v_/F_m_) is very homogeneous (0.55 to 0.75) for well hydrated alcobioses studied (Fig. 5, Fig. S7). Moreover, F_v_/F_m_ for alcobioses and uncovered algal layer does not differ substantially (Fig. 5C,F,I). In contrast, dry systems have F_v_/F_m_ close to zero and recover quickly after rehydration (Figures S7, S8). Minimal fluorescence (F_o_) was increased to some extent when fungal crusts were removed by razor blade from algal layer. It may demonstrate a shielding effect of the fungal partner on algae, particularly in *Lyomyces-Desmococcus* system (Fig. 5J,K,L,M,N,O).

CO_2_ exchange of alcobioses was also very variable but easily detectable, and it confirms the viability and physiological activity of the partners. Typical light response curves of CO_2_ assimilation for five alcobioses, free-living terrestrial alga and foliose lichen are in Fig. S9. The following features were common for all systems: (1) Respiration of just rehydrated alcobioses was much higher (typically five to tenfold) than of those hydrated for a long time, equilibrating to steady-state in hours to days. (2) All alcobioses respond to light, and photosynthesis exceeded overall respiration in some cases (particularly under elevated [CO_2_]). (3) Photosynthesis is saturated under unusually dim light (typically 50 to 100 µmol photons m^−2^ s^−1^) in systems with *Coccomyxa* algae, but may be still increasing in the others, under full sun intensity (ca 1800 µmol m^−2^ s^−1^) and elevated CO_2_, Fig. S5). Moreover, lower temperatures are beneficial for the net carbon gain (Fig. S10).

### Algal carbon is absorbed by fungi in some alcobioses

Pilot (HPLC–MS, GC–MS) experiments did not detect any significant carbon transfer from algal polyols (sorbitol and ribitol) to fungal substances (mannitol and ergosterol) in *Resinicium bicolor-Coccomyxa*, *Skvortzovia furfuracea-Coccomyxa* and *Xylodon asper-Coccomyxa*. In contrast, the control lichens, *Hypogymnia physodes* and *Multiclavula mucida*, expressed a clear pattern of ^13^C transfer from algal ribitol to fungal mannitol after two hours of assimilation in the ^13^C-enriched air. Intermediate results were obtained for *Lyomyces sambuci-Desmococcus* where mannitol expresses some degree of ^13^C enrichment, but barely significant in our set-up (data not shown).

**Figure 6:**
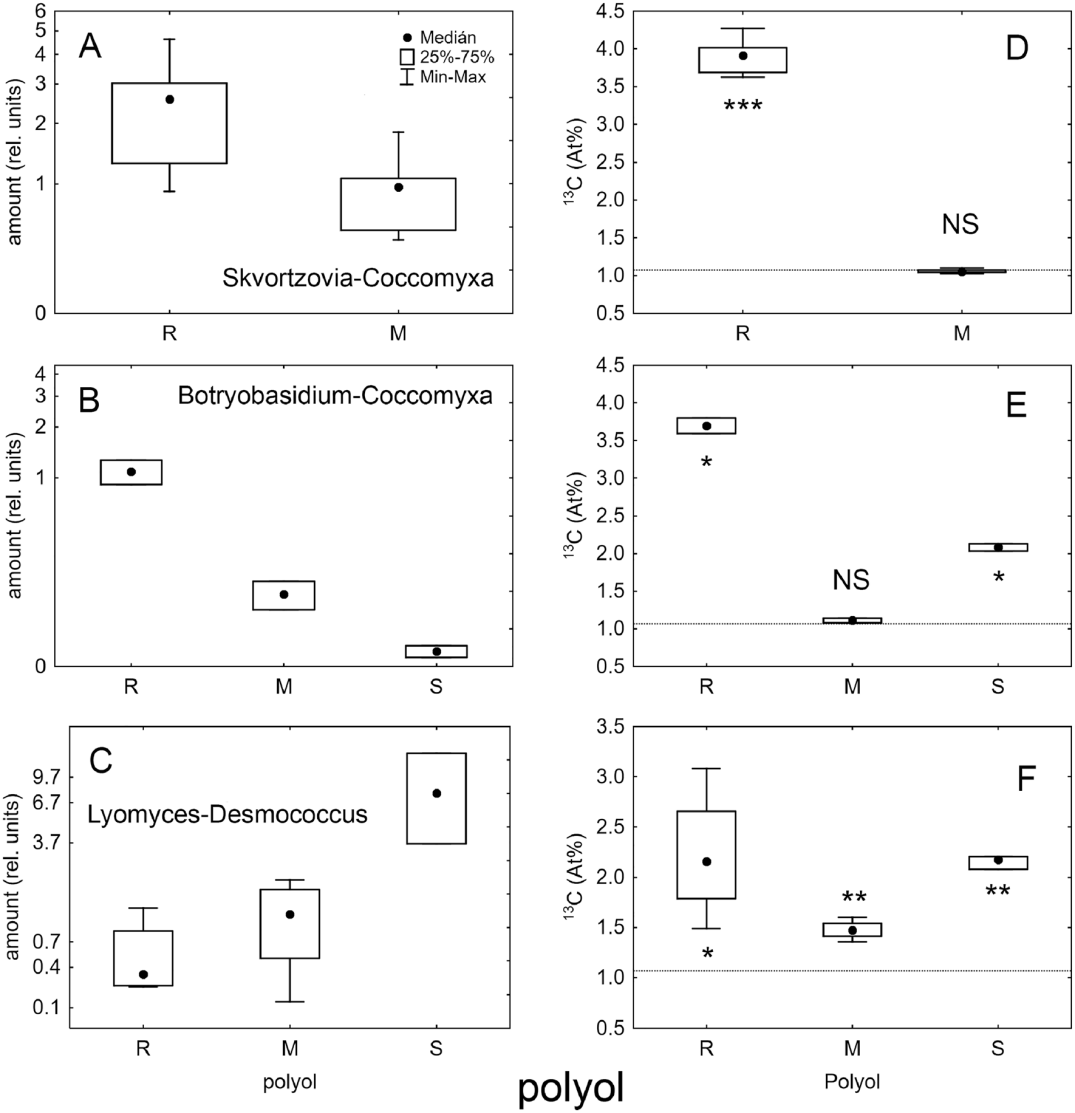
Abundance and ^13^C enrichment in trimethylsilyls of principal algal and fungal polyols after 18 h assimilation of alcobioses in ^13^CO_2_ atmosphere. Algal polyols: ribitol (R) and sorbitol (S); fungal mannitol (M). Median (central point), 25% and 75% quantiles (box) and min–max (whiskers) are shown. Dotted line represents natural ^13^C abundance (1.07 At %) and t-test was performed against it; NS: not significant (*P* > 0.05), *: 0.05 < *P* > 0.01, **: 0.01 < *P* > 0.001, ***: *P* < 0.001.

Subsequent detailed studies using isotope ratio mass spectrometry (IRMS), much more sensitive for isotope abundances, were performed (Method S1). These experiments delivered two strikingly different results: (1) The *Skvortzovia furfuracea-Coccomyxa* system did not display any algal-fungal carbon transport. The principal polyols were algal ribitol and fungal mannitol (Fig. 6A). TMS-ribitol was highly ^13^C enriched (3.90 ± 0.26 At%, t = 24.4, *P* < 0.001, N = 5) after 18 h of labelling whereas the fungal TMS-mannitol remained very close to the natural ^13^C abundance (1.07 ± 0.03 At%, t =  − 0.8, *P* = 0.45, N = 5; Fig. 6D). (2) The algal-fungal carbon transfer was confirmed in the *Lyomyces sambuci-Desmococcus* alliance, where sorbitol is the principal algal polyol (Fig. 6C). We recorded a substantial ^13^C signal in TMS-sorbitol (2.15 ± 0.07 At%, t = 28.8, *P* = 0.001; N = 4) and a slightly lower but still very significant signal in TMS-mannitol (1.48 ± 0.10 At%, t = 8.2, *P* = 0.004, N = 4; Fig. 6F).

Two specimens of *Botryobasidium-Coccomyxa*, measured in parallel, having low biomass and, thus, low polyol abundances (Fig. 6B), delivered very convincing negative result. Its TMS-ribitol ^13^C enrichment was high and consistent (3.70 ± 0.14 At%, t = 25.9, *P* = 0.024; N = 2) but mannitol invariant from natural abundance (1.11 ± 0.04 At%, t = 1.4, *P* = 0.39, N = 2; Fig. 6E), very similar to *Skvortzovia furfuracea-Coccomyxa* system. See also chromatograms (Figures S11, S12).

### Snails rejuvenate alcobioses and produce isidia-like diaspores

**Figure 7:**
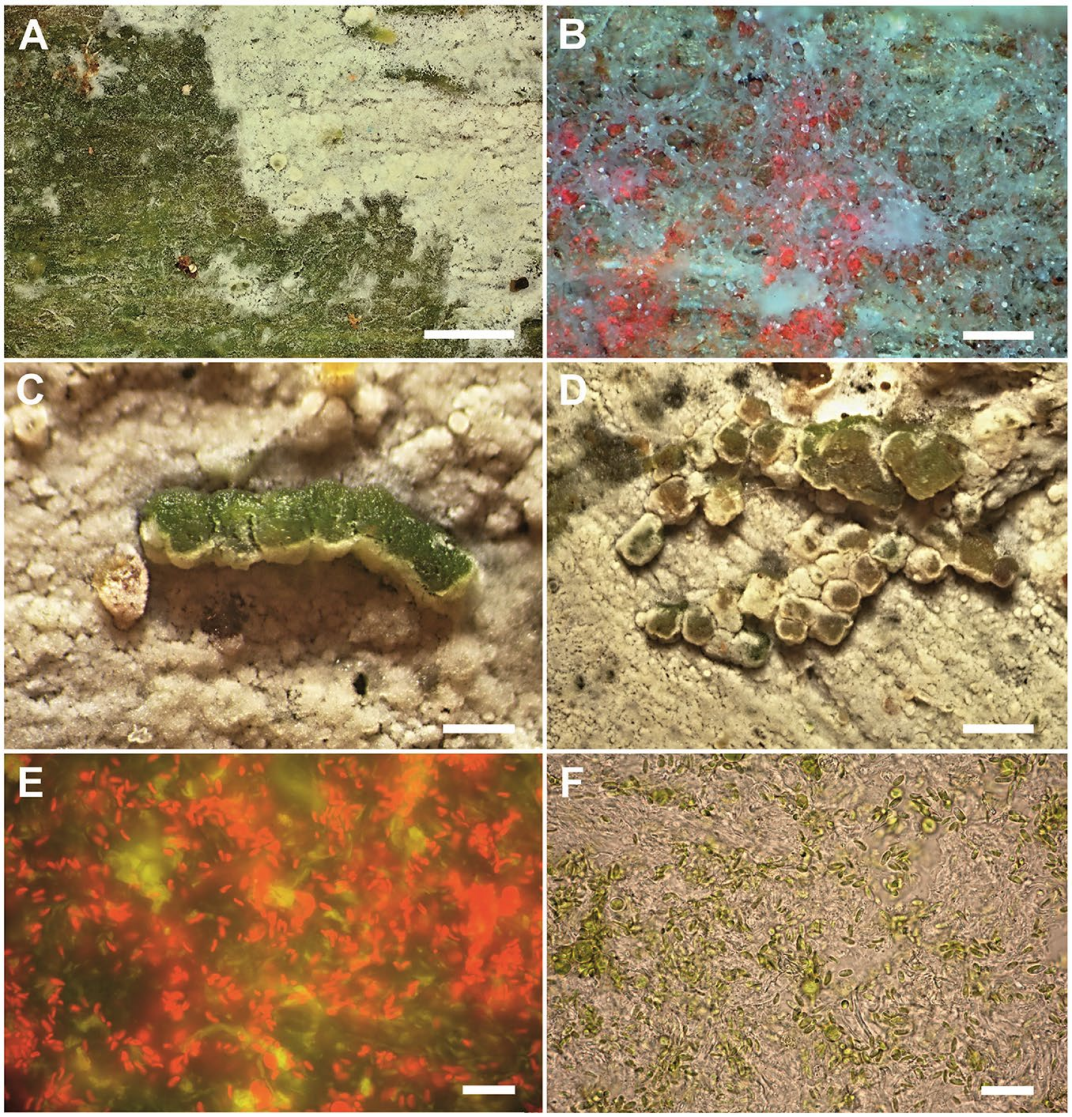
Snail-grazed *Skvortzovia furfuracea*. (**A**) a green area of exposed algal layer formed by snail grazing; (**B**) regenerated mycelium overgrowing grazed areas. Algal cells are indicated by the red chlorophyll autofluorescence. (**C**) fresh snail excrement; (**D**) excrements are quickly overgrown by regenerated mycelium; (**E**) the red chlorophyll autofluorescence indicates high density of *Coccomyxa* cells in an excrement; (**F**) a mixture of *Coccomyxa* and remnants of fungal tissue in an excrement. Scales: (**A**) 2 mm; (**B**) 0 µm; (**C**, **D**) 1 mm; (**E**, **F**) 20 µm.

Literature says little about the persistence of the corticioid fungal crusts involved in this study. According to our field observations, crusts of *Lyomyces sambuci*, *Resinicium bicolor* and *Skvortzovia furfuracea* can persist over several years. Persistence of alcobioses is apparently assisted by snail grazing. Large areas of fungal crusts including algae are frequently removed by snails and the algal layer is uncovered (Fig. 7A). Grazed spots are quickly overgrown by rejuvenated fungal hyphae (Fig. 7B). Therefore, fungal crusts, especially of *Skvortzovia furfuracea*, typically form a continuous mosaic of younger and older (i.e. thinner and thicker) patches.

Snail excrements form distinct structures accompanying alcobioses. They have a granular or caterpillar-like shape (Fig. 7C) and, when lying on grazed fungus, they are quickly overgrown by young hyphae and incorporated into the fungal crust (Fig. 7D). Fresh excrements are green, densely filled by living algal cells (Fig. 7E) in a mixture with remnants of fungal hyphae (Fig. 7F). The algal-fungal content makes excrements an analogous structure to vegetative propagules (e.g. isidia) of lichens and may serve for vegetative reproduction when being placed/translocated outside the current fungal crust. The same function was described at mite excrements that contained viable fungal and algal cells of the lichen *Xanthoria parietina*^25^.

## Discussion

### Corticioid fungi associated with algae

Corticioid fungi are generally considered saprophytic, rarely parasitic and mycorrhizal; the frequent association with algae has largely been ignored in most monographs^26^. However, Parmasto^27^, already in 1967, observed an alcobiosis in the newly described fungus *Phlebia lichenoides*, as he refers to an internal algal layer containing *Chlorococcus*-like cells. Hjortstam et al.^28^ considered *Phlebia lichenoides* as a synonym of *P. subcretacea* (currently *Cabalodontia subcretacea*) and dismissed its association with algae.

Poelt & Jülich^15^ provided the most comprehensive study so far on alcobiosis revealed in *Resinicium bicolor,* and refer to the presence of an algal layer formed, allegedly, of *Coccomyxa glaronensis*. (This algal species was described as a symbiont in the lichen *Solorina saccata*^29^). Poelt & Jülich^15^ observed algae in all surveyed specimens and algal colonies were either restricted below fungal crusts or only slightly expanded out of the fungal spots and formed a surrounding green rim (which corresponds with our observations; Fig. 5A,B). The authors classified the algal-fungal contact as intimate, but without obvious appressoria or haustoria. Subsequent short notes in literature^13,30–32^ have only repeated the findings by Poelt & Jülich. Whereas Oberwinkler speculated in 1970 that *R. bicolor* represents a basidiolichen^30^, he omitted this fungus from his later overview of basidiolichens^33^.

Two related recent conference contributions refer to associations of corticioid *Hyphodontia* s.lat. (namely *Lyomyces crustosus*, *Hyphodontia pallidula* and *Xylodon brevisetus*) with algae *Coccomyxa* and *Elliptochloris*, but without molecular sequence data^16,17^. It is not clear from the published meeting abstracts, if both algal genera were observed co-occurring in an association with a single fungal crust, or if each algal-fungal system involved only a single algal partner. We have only observed the latter case. The anatomical observations reported by Voytsekhovich et al.^17^ allegedly revealed appressoria and haustoria in hyphae attached to algal cells and, on this basis, the authors consider these fungi to be optionally lichenised. Physiological relationships between the corticioid fungi and their algae were not studied. Our data confirmed that some species of *Hyphodontia* s.lat. are frequently involved in alcobioses, but we do not consider them lichens (see below). Whereas *Coccomyxa* is undoubtedly frequent in alcobioses, we did not detect *Elliptochloris* in specimens collected in the current study.

Associations of corticioid fungi and algae sometimes have a typically parasitic character. It was demonstrated for *Athelia epiphyla*^15^ where fungal hyphae form haustoria penetrating algal cells. This fungus causes bleaching of corticolous algae, i.e. forms characteristic pale-grey rounded spots on otherwise green tree bark. Although *A. epiphyla* is apparently a parasite, Jülich^34^ and Oberwinkler^33^ refer to symbiotic relationships between epiphytic algae and some species of *Athelia* and *Athelopsis*. Some of these cases may be close to our concept of alcobiosis.

Parallel research has been conducted on associations of polypore fungi and their epiphytic algae. The surface of polypore fruiting bodies is a suitable substrate for numerous algal (and cyanobacterial) species^35,36^. However, these associations do not show any specificity–a single polypore is often covered by a community of several algal species with broader niches (not restricted to polypores). Carbon transfer from epiphytic algae to polypore fungal tissues was repeatedly reported using ^14^C tracer^37,38^, but we are skeptical of that result as the algae usually grow on/in dead polypore tissues. Thus, this association seems to be very different from alcobioses with algae embedded within the viable fungal tissue.

The taxonomic composition of algae found in alcobioses is in general not surprising. Unicellular trebouxiophyte algae such as *Coccomyxa* and *Stichococcus* sensu lato are ubiquitous in terrestrial habitats and have been long known to create various types of fungal-algal consortia^39^. In our study we employed molecular barcoding of the algal partners in alcobioses for the first time, which allowed us to place all three detected taxa into narrowly defined and strongly supported monophyletic clades of *Coccomyxa* sp., *Desmococcus olivaceus*, and *Tritostichococcus coniocybes*. Each of these clades probably corresponds to a single algal species, judging from their uniform morphology and high *rbcL* gene sequence identity (Fig. 4, Figures S3, S4, S5). The *Coccomyxa* sp. found in our samples evidently represents a species new to science, but its transfer to pure culture, deposition of a holotype, and formal description was beyond the scope of the current study. As well as living in alcobioses, we know from previously published sequences that each of these three genospecies can live as a free-living terrestrial alga^23,24^. In the case of *D. olivaceus*, our results are the first reliable observation of this species in symbiotic association with fungi, but *S. coniocybes* and the particular clade of *Coccomyxa* have previously been reported as genuine lichen photobionts^22,24^.

### Ecophysiological implications

We confirmed the viability of algae in alcobioses via chlorophyll *a* fluorescence^40^ and gas exchange measurement. The maximal quantum yield of photosystem II (F_v_/F_m_) is a well-accepted measure of vitality and impact of stress. In the case of alcobioses, F_v_/F_m_ confirms that the algae are not stressed under the fungal layer, having F_v_/F_m_ comparable to values for adjacent free-living algae (Fig. 5C,F,I). Photochemistry recovered within minutes to tens of minutes to values > 0.4 after dry alcobioses were rehydrated (Figures S7, S8), being considered “physiologically active”, in accordance with studies on terrestrial algae^41^.

The minimal fluorescence (F_o_), in turn, shows a low shielding effect of fungal crusts to incident light, particularly for shade-adapted alcobioses formed by *Coccomyxa* and fungi with rather thin basidiomata (Fig. 5J,K,L,M). A substantial shielding effect was detected at the thicker and more light-scattering crust of *Lyomyces sambuci*, growing in more-light exposed sites, which may be photoprotective for *Desmococcus* algae in this association (Fig. 5 N,O).

The CO_2_ exchange, reflecting the real photosynthetic performance and production process, has not been studied in alcobioses yet, but reference data are available for lichens^42–44^. Here we provide gas-exchange data for four alcobiosis systemes in the context of lichen symbiosis (represented by *Parmelia sulcata*) and a free-living algal crust (*Trentepohlia aurea*). Figures S9 and S10 demonstrate relationships of the CO_2_ exchange to an incident light intensity, gaseous CO_2_ concentration and ambient temperature. Respiration of the fungal partner plus co-occurring microbiomes was usually higher in shade-adapted *Coccomyxa*-based alcobioses (Fig. S9). Thus, net carbon balance of the system was mostly negative and algal photosynthesis could not serve as a principal source of carbon for the fungus. Saturating light intensity was also unusually low (50 to 100 µmol photons m^−2^ s^−1^) suggesting a strong shade adaptation of the algae involved. In contrast, free-living *Trentepohlia aurea,* the lichen *Hypogymnia physodes* and the alcobiosis *Lyomyces sambuci-Desmococcus* occurring in more light-exposed conditions, had higher maximal CO_2_ assimilation and higher light saturation intensity and the carbon gain of these systems was frequently positive. CO_2_ assimilation rate rising even close to full sun intensity under elevated CO_2_ suggests high diffusional limitation of the photobionts and their high photosynthetic capacity as well (Fig. S9). In addition, the overall carbon balance of those systems is strongly temperature dependent. Lower temperatures promote carbon gain of alcobioses, as demonstrated on *Xylodon* system (Fig. S10). This can be explained by higher temperature dependency of dark respiration (R_dark_) than gross photosynthetic capacity (A_gross_). Comparable magnitudes of R_dark_ and A_gross_ will lead to substantial effect of temperature on carbon gain^45^.

We observed a significant alga-to-fungus ^13^C transfer in only one of the systems studied. We selectively measured algal (ribitol, sorbitol) and fungal (mannitol) compounds. Polyols are not suitable for gas chromatography, therefore their trimethylsilyls (TMS-) were measured^46^. That is, the five-carbon ribitol was converted to the twenty-carbon TMS-ribitol (C_20_H_52_O_5_Si_5_). Similarly, six-carbon mannitol and sorbitol led to the formation of 24-carbon TMS-derivatives (C_24_H_62_O_6_Si_6_). Thus, the ^13^C enrichment (indicating carbon transfer) is four-times more pronounced in mother molecules than in TMS derivatives measured here and thus the negative result for *Skvortzovia furfuracea-Coccomyxa* is very convincing (Figs. 7 and S11). The isotopic precision of IRMS is better than 0.01 At% of ^13^C for isotope ratios close to natural (< 5 At% of ^13^C). Mannitol ^13^C is clearly unchanged from its natural value in this system, whereas TMS-ribitol is highly enriched up to 4.6 At% ^13^C (by 3.5 At% compared to natural). This means that the parent ribitol should have up to 15.1 At% ^13^C (4 × 3.5 + 1.1). In contrast, the *Lyomyces-Desmococcus* TMS-mannitol was significantly enriched by approximately 0.4 At% (Figs. 7 and S12). Thus, mannitol should be enriched up to 2.7 At% (1.1 + 4 × 0.4 At%). Despite the clear statistical significance, more work is needed to decipher the timing, environmental dependencies and, thus, ecological significance of those carbon transfers.

### Symbiosis on the threshold of lichenisation

The lichen is defined above in the Premise 2 as a symbiosis of alga or cyanobacterium (photobiont) and fungus (mycobiont) with following specifics: (1) The mycobiont is nutritionally dependent on its photobiont^47^. (2) The mycobiont is not obviously harmful to its photobiont^48^. (3) The photobiont occurs within the mycobiont thallus^49^. (4) Mycobionts and photobionts usually cannot persist over a long period outside the symbiosis^50,51^.

Alcobioses do comply with points (2) and (3). They have an internal lichen-like algal layer (Figs. 1, 2, 3) and the algae thrive in the symbiosis (Fig. 5, Figures S3, S4, S5, S6). Only *Lyomyces-Desmococcus* partly complies with point (1); we confirmed carbon transfer from alga to fungus (Fig. 6, Figures S11, S12). We suggest however that *Lyomyces* is not fully dependent on algal assimilates, because it has been observed occasionally without the algal symbiont. Consequently, *Lyomyces-Desmococcus* does not meet point (4), because both symbionts may live apart. Surprisingly, *Desmococcus olivaceus* is absent from the list of lichen photobionts^39^ and is mostly reported free-living, forming extensive epiphytic or epilithic algal crusts^52^. Another member of the genus, *Desmococcus vulgaris*, was found overgrowing fruiting bodies of the polypore *Fomes fomentarius*^53^. *Lyomyces sambuci*, like other *Hyphodontia* s. lat., is believed to cause white rot, i.e. is saprophytic and, remarkably, the frequently present algal layer in this common fungus was not mentioned or illustrated in the monographs^26,28^. Point (4) is partly met in some alcobioses with the *Coccomyxa* species known from lichens^9,22^. At our sampling sites, the alga appears to occur only inside and below the fungal crusts (Fig. 5A,B). Contrary to our observations, an almost identical genotype of *Coccomyxa* was found free-living on the bark of live pine and oak trees in the study of corticolous algae by Kulichová et al.^23^. Therefore, the lifestyle of this alga seems to be diverse, and investigations on the population level are needed to elucidate the potential specific life strategies of individual strains. Fungi associated with *Coccomyxa* were observed to live without algae, but the alcobiosis is almost omnipresent in *Resinicium bicolor* and *Skvortzovia furfuracea*. The nature of this symbiosis remains enigmatic as it probably has no nutritional character.

In conclusion, the photosynthetic potential of algal partners is clearly substantial, but their direct nutritional importance for fungi (and whole alcobioses) is still obscure, even in *Lyomyces-Desmococcus,* and needs more study. Simultaneously, the insignificant carbon exchange observed in most systems implies that there must be other ecological advantages keeping the partners in a stabile association (e.g. exchange of bioactive substances under stress conditions such as drought).

## Materials & methods

In the period 2016–2021, we recorded 58 specimens with alcobiosis (Table S1). Corticioid fungi were identified to species from their morphology and 27 specimens were barcoded by ITS nrDNA sequences. Algae were determined to genus according to their morphology and 20 specimens were barcoded by sequencing the ribulose bisphosphate carboxylase large subunit gene (*rbcL*). The NCBI accession numbers of the sequences obtained are provided in Table S1. Field collections from 2019–2021 were employed in morphological and anatomical observations (fluorescent microscope Olympus BX 61 in bright field and fluorescent mode) and in physiological measurements that were done within one week after collection. Before that, samples were hydrated if necessary, put in Petri-dishes and accommodated in LED illuminated cultivating room under 20 °C, irradiation about 100 µmol m^−2^ s^−1^ and photoperiod of 12 h for at least two days.

### DNA barcoding & phylogenetic analysis

Fresh specimens were used for DNA extraction of both fungal and algal partners. DNA was extracted with a cetyltrimethylammonium bromide (CTAB)-based protocol^54^. ITS nrDNA locus was found to be a useful fungal barcode sequence, easily amplifiable, and moreover with a sufficient number of references in the NCBI database. In the case of algae, we sequenced nrDNA ITS and 18S regions and the *rbcL* gene. The latter was found as best amplified and therefore selected for barcoding and phylogenetic analysis. Polymerase chain reactions were performed in a reaction mixture containing master mix consisting of 2.5 mmol/L MgCl_2_, 0.2 mmol/L of each dNTP, 0.3 μmol/L of each primer, 0.5 U Taq polymerase in the manufacturer’s reaction buffer (Top-Bio, Praha, Czech Republic), and milli-Q water to make up a final volume of 10 μL. The primers used for PCR and the cycling conditions are summarized in Table S2. Successful amplifications were sent for Sanger sequencing (GATC Biotech, Konstanz, Germany). Sequences were edited using BioEdit v.7.0.9.0^55^ and Geneious Prime 2022.0 (https://www.geneious.com).

Sequences of our specimens were supplemented by relevant sequences from GenBank—NCBI database. Sequences were aligned by MAFFT v.7^56^; available online at http://mafft.cbrc.jp/alignment/server/) using the Q-INS-i algorithm and adjusted manually. The best-fit model of sequence evolution was selected using the Akaike information criterion calculated in jModelTest v.0.1.1^57^. Relationships were assessed using Bayesian inference as implemented in MrBayes v.3.1.2^58^. Two runs starting with a random tree and employing four simultaneous chains each (one hot, three cold) were executed. The temperature of a hot chain was set empirically to 0.1, and every 100th tree was saved. The analysis was considered to be completed when the average standard deviation of split frequencies dropped below 0.01. The first 25% of trees were discarded as the burn-in phase, and the remaining trees were used for construction of a 50% majority consensus tree.

### Gas exchange

CO_2_ exchange was measured using the portable photosynthetic system LI-6400XT (Li-Cor, Lincoln, NE, USA) connected to a custom-made peltier-conditioned gas exchange chamber described elsewhere^59,60^, see also Figure. S1. The chamber allows to accommodate samples up to 64 cm^2^ of ground area and 1 cm in thickness. It is of high sensitivity, as we have demonstrated in the past by successfully measuring the very small gas exchange of moss capsules^61^. Algae-containing basidiomata were attached to the razor-thinned native substrate to minimise microbial respiration. “White” PAR irradiation was supplied by a LI6400-18 RGB system source controlled by LI-6400XT. Temperature was maintained at 20 °C (except when measuring the response to varying temperature) and airflow at 250 µmol mol^−1^. The light response of CO_2_ assimilation was measured in continual-logging mode (each 5 s), at least 3 min in each light intensity. After a steady state was reached, data were collected, and light intensity changed to the next value. The temperature curve relied on Peltier-cooling of the cryptogamic chamber. At least 15 min was allowed to pass before data on gas exchange was taken, to allow equilibrium to be reached. Finally, background (empty chamber response) was measured and subtracted.

### Chlorophyll a fluorescence imaging

2D fluorescence measurements were performed by the FluorCam FC800 instrument (PSI, Drásov, Czech Republic). Red LEDs (λ = 660 nm) were used for both, measuring light (10, 20 or 33 µs flashes, in average < 1 µmol m^−2^ s^−1^) and actinic light (continuous, photosynthesis driving, about 100 µmol m^−2^ s^−1^) photon sources. White LEDs (≈ 2500 µmol m^−2^ s^−1^) delivered saturation pulses of 1 s in duration. The camera has 720 × 560 px resolution, 12-bit data depth and is equipped with a zoom objective imaging frame down-to *ca* 10 × 7.5 cm (≈ 0.13 mm per pixel).

### Metabolite transfer measurement

Stable carbon ^13^C was chosen to trace photobiont assimilated carbon. Substrate-attached sporocarps with active algal partner (Li-6400XT-proved, Figure. S1) were enabled to assimilate ^13^CO_2_ enriched air in a water-sealed 3L inverse Petri dish with internal fan (See Figure. S2). The labelling device is described in detail elsewhere^61^. [^13^CO_2_] was about 1000 µmol mol^−1^. Atmospheric CO_2_ was previously replaced by CO_2_ free synthetic air and then ca 3 mL of ^13^CO_2_ (> 99 atom %, Sigma-Aldrich, Luis., USA) was injected. Samples assimilated for two to 18 h under about 200 µmol m^−2^ s^−1^ of white LED light. Then fungal/algal crusts were scratched down by razor and killed in boiling methanol. Homogenised and filtered metOH extracts were used for analysis of polyols and ergosterol (see Methods S1 for further details).

We employed three approaches to trace ^13^C enriched metabolites. (1) HPLC–MS to separate algal polyols (ribitol and sorbitol) from fungal mannitol and to measure their ^13^C content. Although mannitol was detected in some algal groups^62,63^, it is not produced by algae in our systems, i.e., *Coccomyxa* and *Desmococcus*^64^. (2) GC-IRMS to separate trimethylsilyl derivatives of those polyols (TMS-ribitol, TMS-mannitol and TMS-sorbitol) and, again, to quantify their ^13^C enrichment. And (3) HPLC–MS to separate fungal specific ergosterol and to measure if it is ^13^C enriched. For more details see Method S1.

## Supplementary Information

Supplementary Information.

## Data Availability

The datasets generated during and/or analysed during the current study are available from the corresponding author on reasonable request.
