# Supplementary material for: Alcobiosis, an algal-fungal association on the threshold of lichenisation
Source: Sci Rep. 2023 Feb 28;13:2957. doi: 10.1038/s41598-023-29384-4 (PMC9975235; doi:10.1038/s41598-023-29384-4)
Supplement: Supplementary file 1 — Supplementary Information. [file 41598_2023_29384_MOESM1_ESM.docx]

Article title: **Alcobiosis, an algal-fungal association on the threshold of lichenisation.**

Authors: Jan Vondrák, Stanislav Svoboda, Lucie Zíbarová, Jan Mareš, Lenka Štenclová, Václav Pouska Jiří Košnar^1,2^ &Jiří Kubásek^3^

Article acceptance date: Click here to enter a date.

**The following Supporting Information is available for this article:**

**Fig. S1 Gas exchange set-up and detail of the cryptogamic gas exchange chamber.**

**Fig. S2 ^13^CO_2_ labelling facility.**

**Fig. S3 Phylogenetic tree of corticioid fungi involved in alcobioses based on ITS sequence data.**

**Fig. S4 Phylogenetic tree of *Coccomyxa* and related taxa based on rbcL sequence data.**

**Fig. S5 Phylogenetic tree of *Stichococcus* and related taxa based on rbcL sequence data.**

**Fig. S6 Phylogenetic tree of *Coccomyxa* and related taxa based on rbcL sequence data.**

**Fig. S7 Recovery of primary photosynthesis of dry *Skvortzovia furfuracea-Coccomyxa* alcobiosis after remoistening**

**Fig. S8 Maximal quantum yield (F_v_/F_m_) of several alcobioses.**

**Fig. S9 Typical light responses of CO_2_ assimilation for five alcobioses, terrestrial alga and lichen under ambient and elevated CO_2_ concentration.**

**Fig. S10 Temperature photosynthetic curve of *Xylodon paradoxus* alcobiosis.**

**Fig. S11 GC-IRMS analyses of trimethylsililated ribitol (RT=388s) and mannitol (436s) in *Skvortzovia furfuracea-Coccomyxa* alcobiosis after 18 h assimilation in ^13^CO_2_ atmosphere**

**Fig. S12 GC-IRMS analyses of trimethylsililated ribitol (RT=925s), mannitol (1550s) and sorbitol (1575s) in *Botryobassidium botryosum-Coccomyxa* and *Lyomyces sambuci-Desmococcus* after 18 h assimilation in ^13^CO_2_ atmosphere.**

**Methods S1 Metabolomics: Gas chromatography – mass spectrometry**

**Table S1 Specimens of investigated alcobioses (uploaded as a separate file).**

**Table S2 Loci used in this study**

**Fig. S1 Gas exchange set-up (top) and detail of the cryptogamic gas exchange chamber (bottom).** The gas exchange facility is built around a portable photosynthesis system LI-6400XT: Sample chamber is stainless steel, circular, 9 cm in internal diameter (64 cm^2^ ground projection area) with acrylic glass lid. The bottom of the chamber is made from 10-cm thick aluminium plate that works as a heat exchanger between the chamber and Peltier coolers on the opposite site of the plate. The chamber has four housings, two of them are sealed in our design. Additional to the Peltier-stabilised temperature of the chamber, low airflow of 150 µmol air s^–1^ increasing resolution and a by-pass valve to correct for offsets were used.


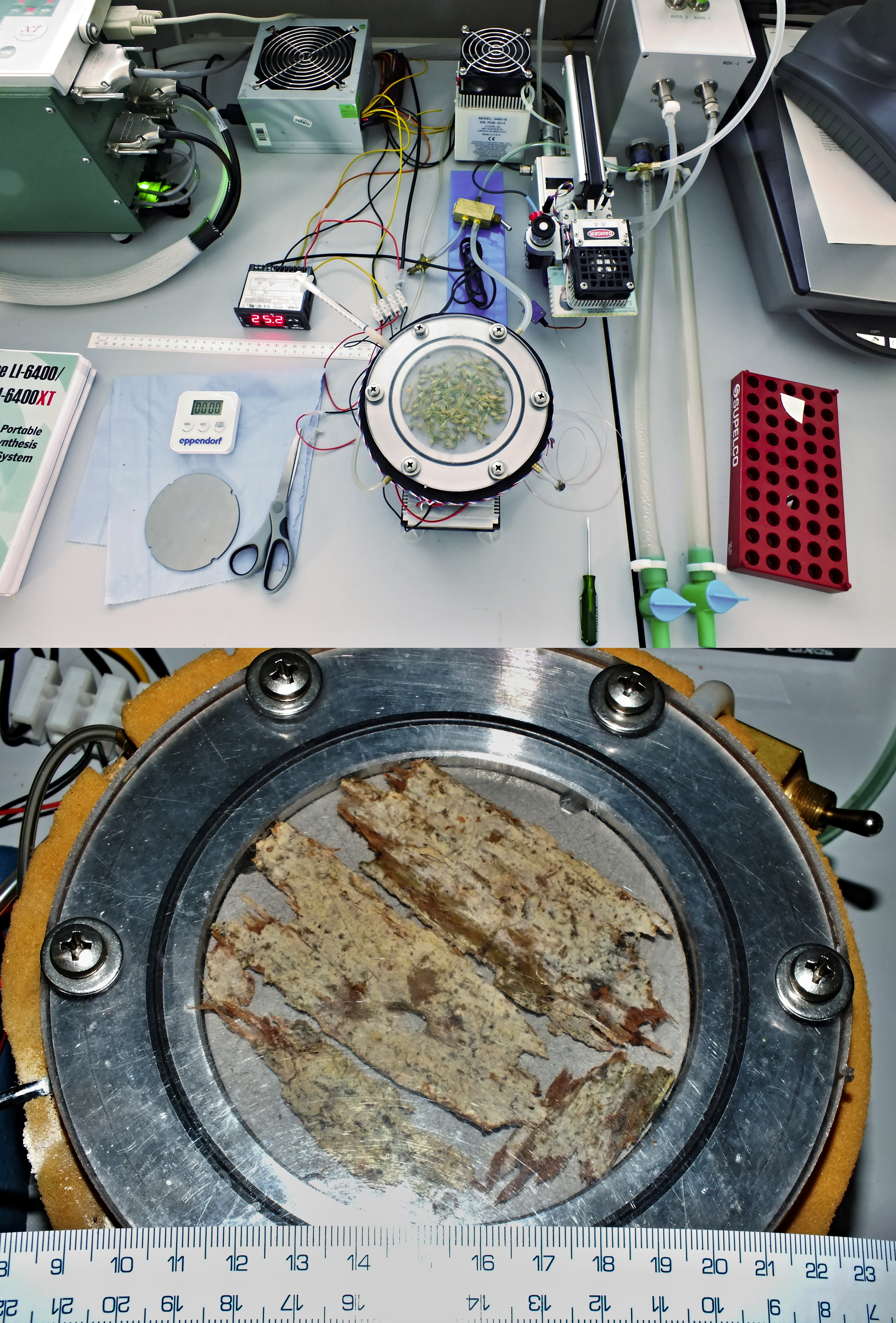


**Fig. S2 ^13^CO_2_ labelling facility.** Inverted, water sealed, 3-L (30 cm in diameter) Petri dish was used in ^13^CO_2_ labelling experiment. Stainless steel 1/16 inch capillary passing the water seal enabled (1) flushing the internal volume by synthetic air to eliminate atmospheric CO_2_ and (2) to inject 3 mL of ^13^CO_2_ to reach ≈ 1000 ppmV [^13^CO_2_]. Miniature fan inside maintained homogeneous internal environment with negligible boundary layer resistance. Virtually 100% internal RH prevented sample desiccation. Intact samples were assimilating under 200 µmol photons m^–2^ s^–1^ supplied by white (4000 K) LEDs and at 20 °C. [CO_2_] did not change more than *ca* 30 % during labelling (determined previously with natural CO_2_).


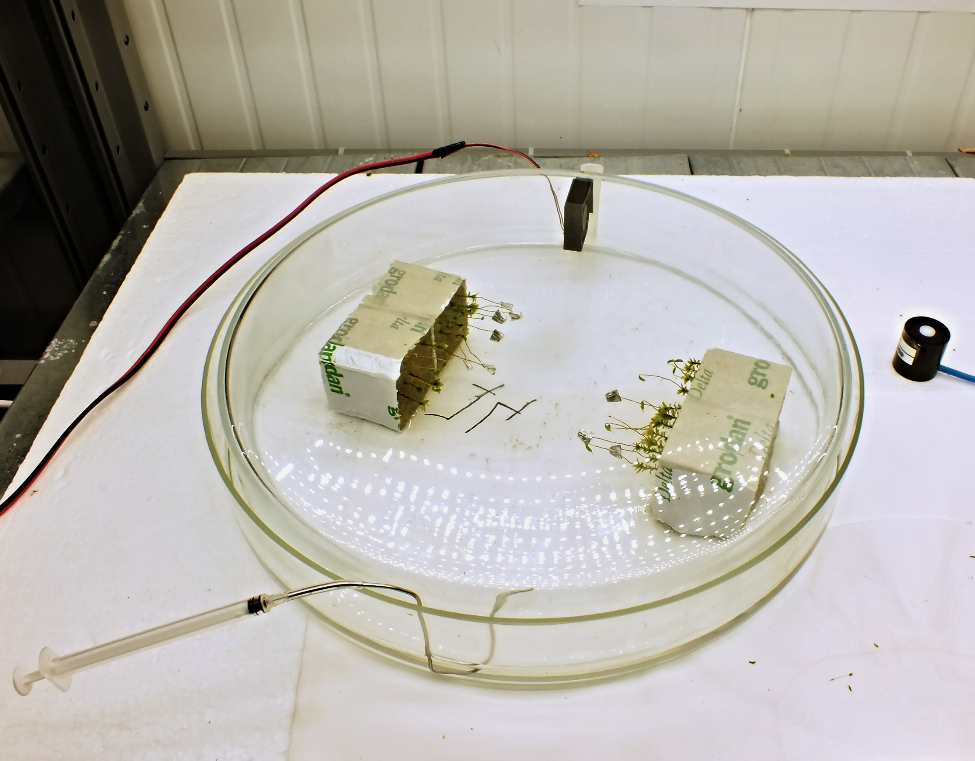


**Fig. S3**  Phylogenetic tree of corticioid fungi involved in alcobioses based on ITS sequence data. The tree was constructed using Bayesian inference involving GTR+G substitution model and was rooted with *Erysiphe alphitoides*. Numbers on branches indicate posterior probabilities. Bold lines indicate branches with posterior probabilities >0.95.


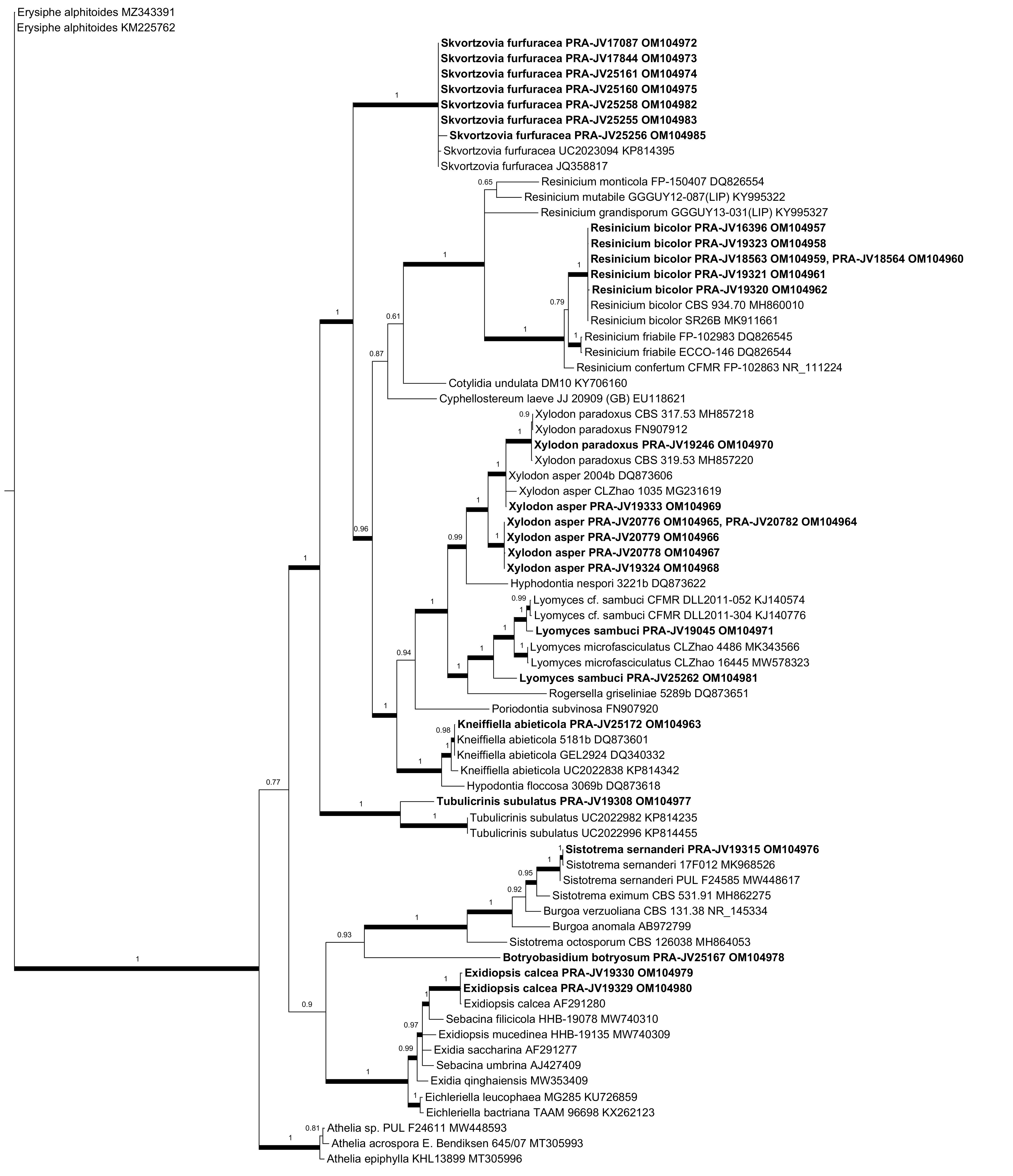


**Fig. S4**  Phylogenetic tree of *Coccomyxa* and related taxa based on rbcL sequence data. The tree was constructed using Bayesian inference involving GTR+I+G substitution model and was rooted with *Elliptichloris*/*Hemichloris* clade. Numbers on branches indicate posterior probabilities. Bold lines indicate branches with posterior probabilities >0.95.


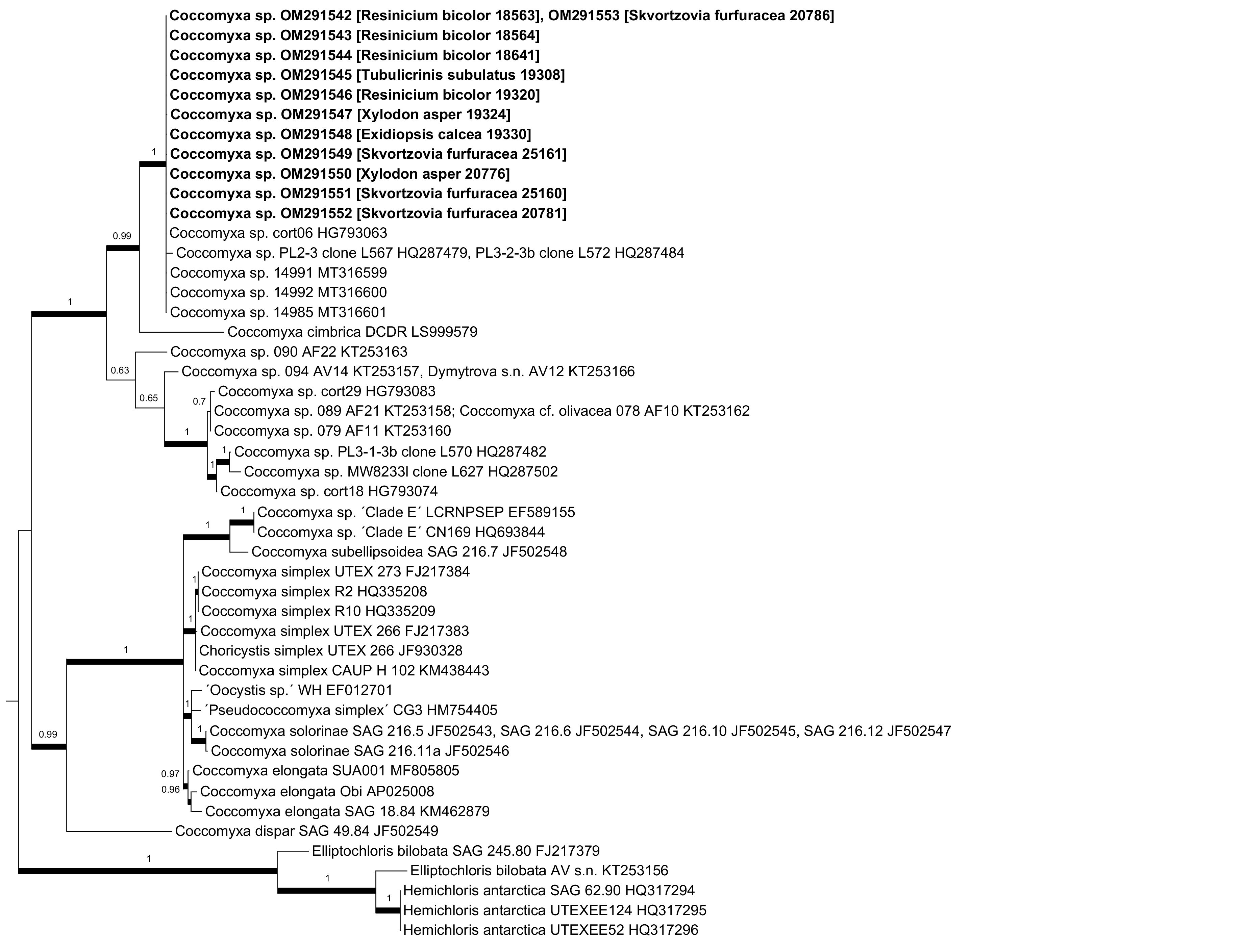


**Fig. S5** Phylogenetic tree of *Stichococcus* and related taxa based on rbcL sequence data. The tree was constructed using Bayesian inference involving GTR+G substitution model and was rooted with *Raphidonema*/*Pabia* clade. Numbers on branches indicate posterior probabilities. Bold lines indicate branches with posterior probabilities >0.95.


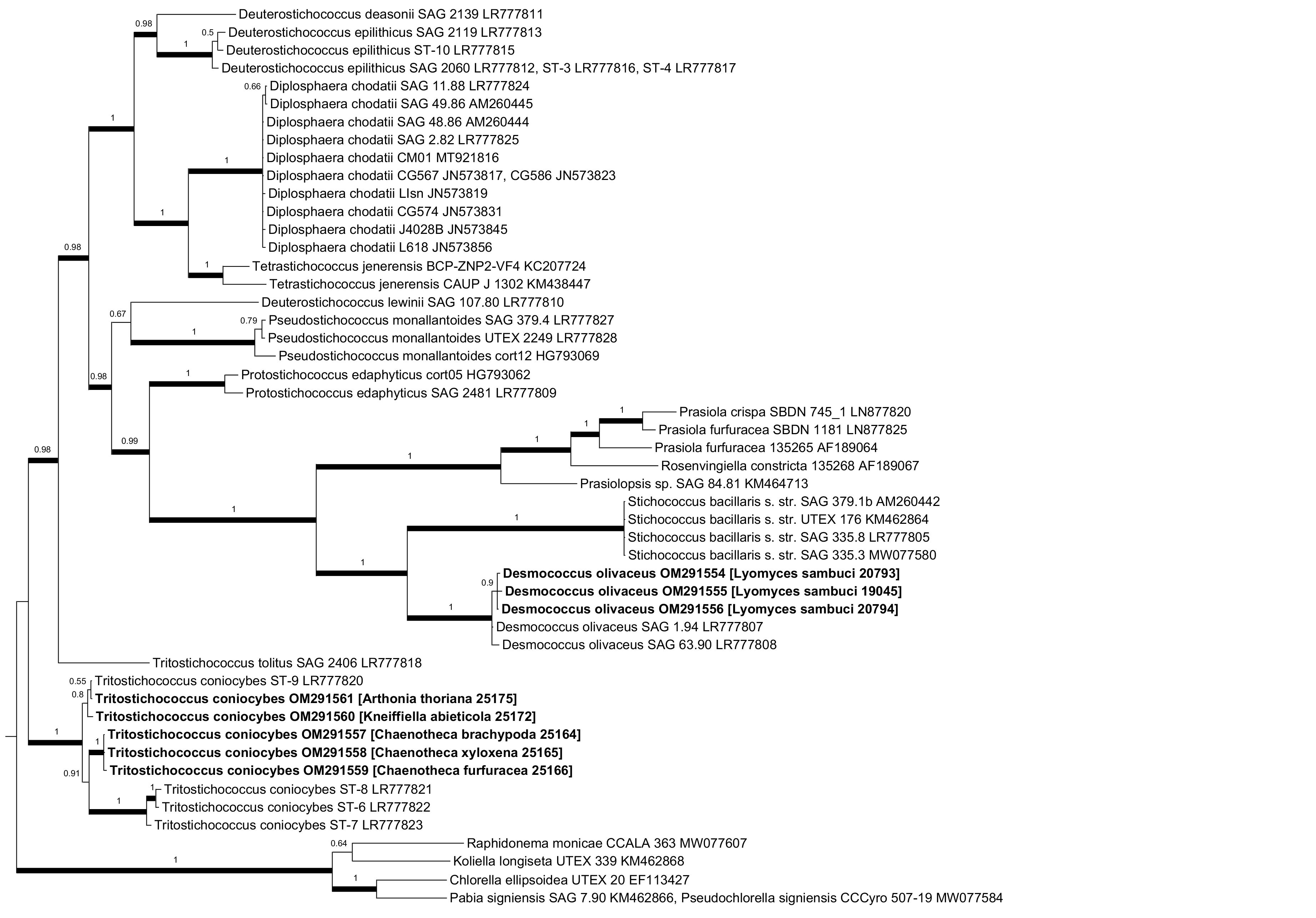


**Fig. S6** Detailed morphology of three algal species detected in alcobioses. A-D: Coccomyxa sp. in Skvortzovia furfuracea (20786). E-H: Desmococcus olivaceus in Lyomyces sambuci (20793). I-L: Tritostichococcus coniocybes in Kneiffiella abieticola (25172). The scalebar represents 20µm in A-H and 10µm in I-L.


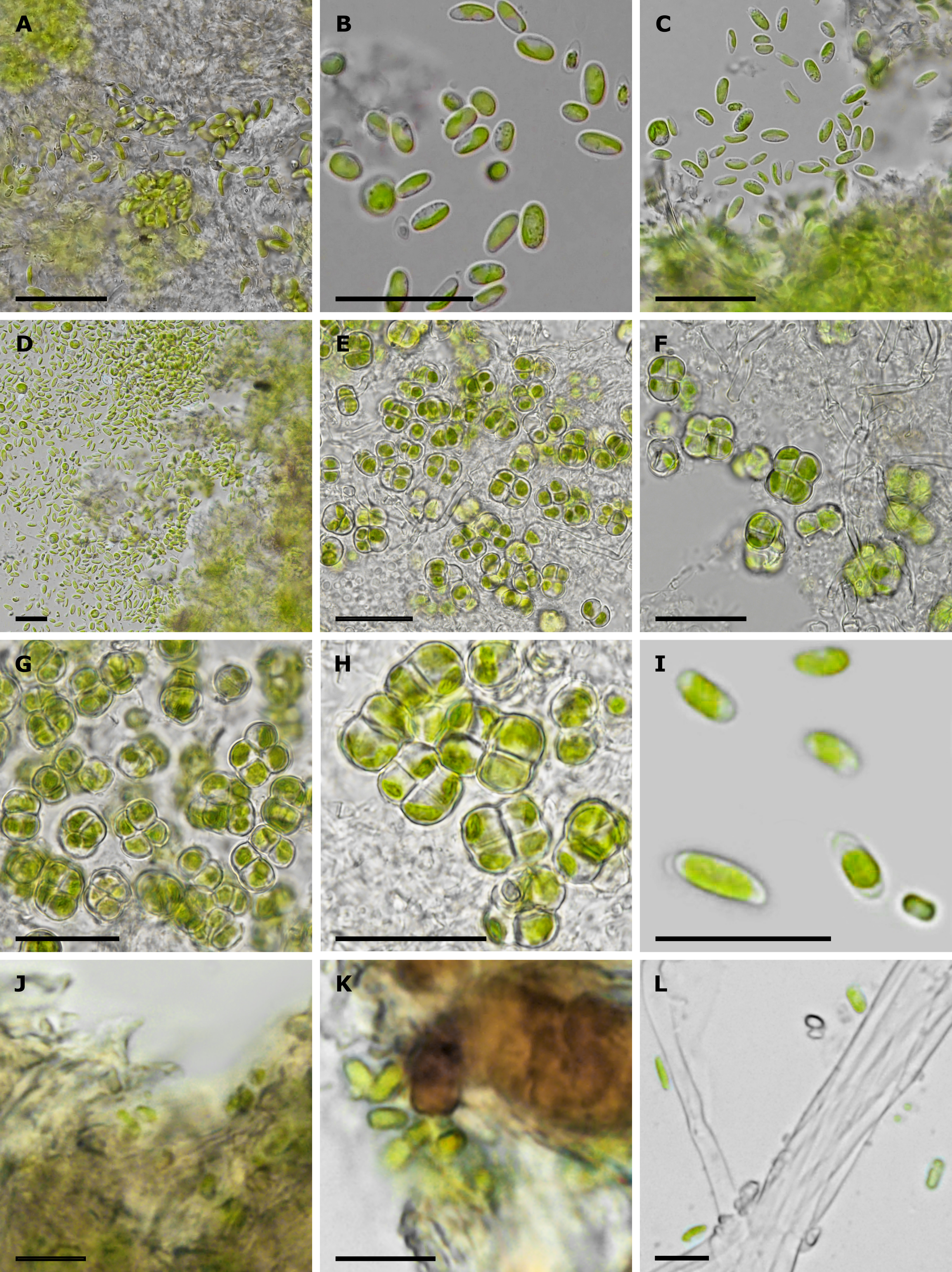


**Fig. S7 Recovery of primary photosynthesis of dry *Skvortzovia furfuracea-Coccomyxa* alcobiosis after remoistening.** Minimal (F_o_, left column), Maximal fluorescence (F_m_ , middle column) and maximal quantum yield of photosystem II (F_v_/F_m_, right column) are shown for dry specimen, just moistened and further times after that. Fungal crust (probably delaying rehydration) boundary is visible only very shortly after remoistening (about 1 min), particularly at F_m_. F_v_/F_m_ recovers to mediate values very quickly (0.47 average 5 min of rehydration) but needs tens of min to reach steady state (typically 0.55 to 0.75 for samples measured). Last time point (120 min) shows beginning of desiccation in the middle of the specimen. The squares below is fluorescence standard without variable fluorescence (F_v_/F_m_ should be close to zero).


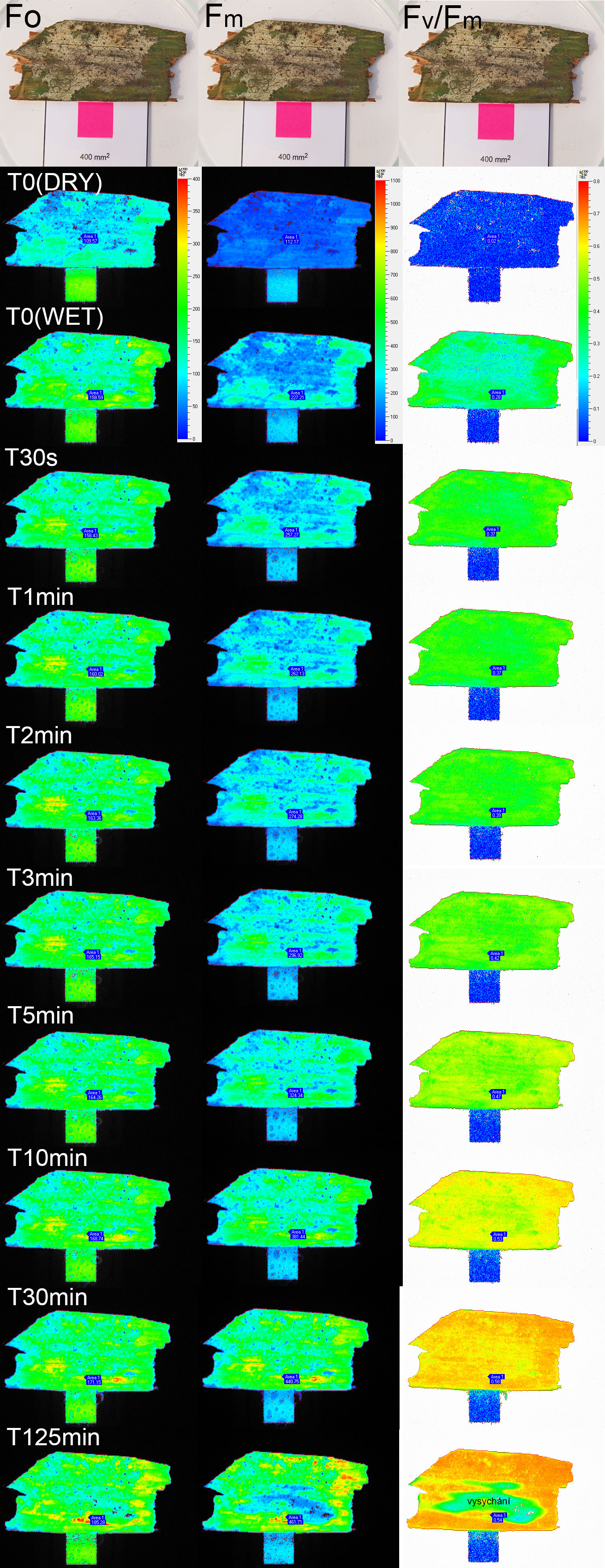


**Fig. S8 Maximal quantum yield of photosystem II (F_v_/F_m_) of several replications of *Skvortzovia furfuracea* –*Coccomyxa* (“Resfur”), *Resinicium bicolor* - Coccomyxa (“Resbic”) and control basidiolichen *Multiclavula mucida* (“Mult High” and “Mult Low”).** Dry samples have F_v_/F_m_ close to zero whereas specimens hydrated for sufficient time (> 60min) have very homogeneous values between 0.55 and 0.7 despite free algae visible on the substratum or covered by fungal crust. In the same time, values are comparable to true lichen – *Multiclavula mucida*.


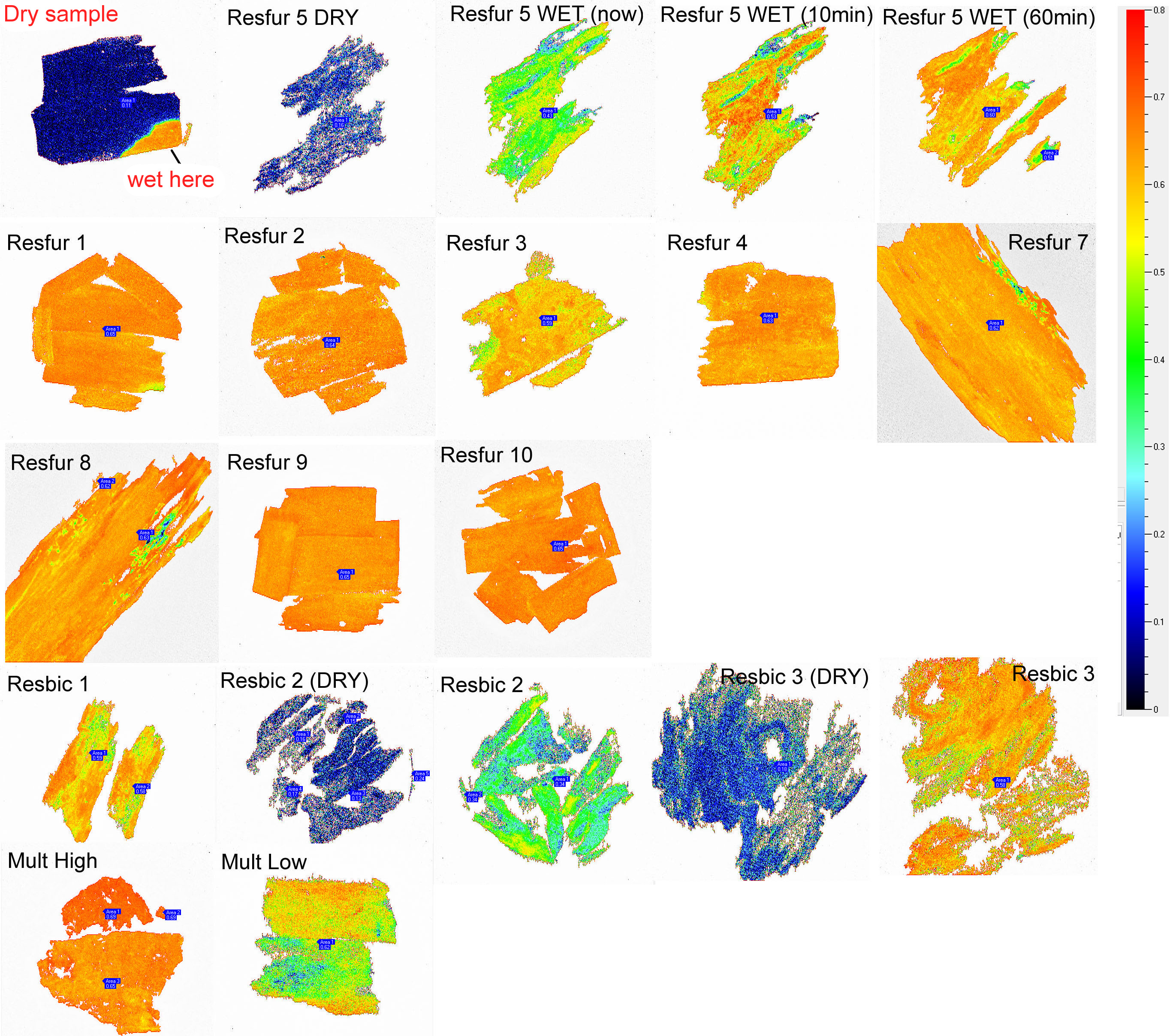


**Fig. S9 Typical light responses of CO_2_ assimilation for five alcobioses, terrestrial alga and lichen under ambient (upper) and elevated CO_2_ concentration (bottom).** Alcobioses growing in deep understory of forests (*Tubulicrinis subulatus-Coccomyxa*, *Resinicium bicolor-Coccomyxa* and *Xylodon asper-Coccomyxa*) have very low photosynthetic capacity just recycling respiratory CO_2_ even under five-times elevated its atmospheric concentration. In the same time, photosynthetic capacity of algae are saturated in low to very low light intensity (< 100 µmol m^-2^ s^-1^ in case of *R. bicolor*). *Lyomyces sambuci*-*Desmococcus* with more sunny habitats have higher photosynthetic capacity and whole alcobiosis may be in net carbon gain at least under elevated [CO_2_] and sufficient light intensity. Free-living terestrial alga (*Trentepohlia aurea*) and lichen *Parmelia sulcata* have the highest photosynthetic capacity and low compensation irradiance (light threshold when photosynthesis just compensate dark respiration).The samples had area between 20 and 60 cm^2^ (chamber diameter is 9 cm). See also Figure S1 for more info about cryptogamic gas exchange chamber and overall set-up.


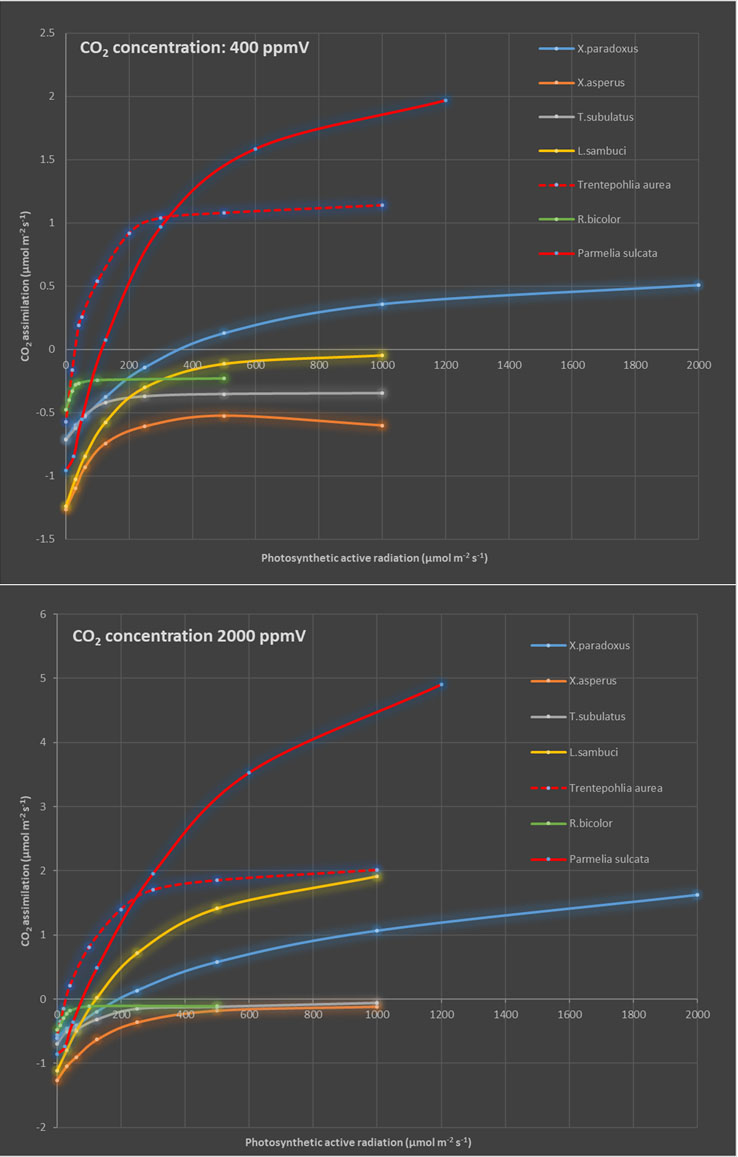


**Fig. S10 Relationship of dark respiration (R_dark_), gross CO_2_ assimilation (A_gross_) and net CO_2_ assimilation (A_max_, A_gross_ - R_dark_) and ambient temperature for *Xylodon paradoxus* alcobiosis under ambient CO_2_ concentration and incident irradiation of 400 µmol m^-2^ s^-1^.** Due to higher effect of temperature on respiration rate than photosynthesis, net carbon gain of this system is sustained only when temperature is lower than about 19 °C.


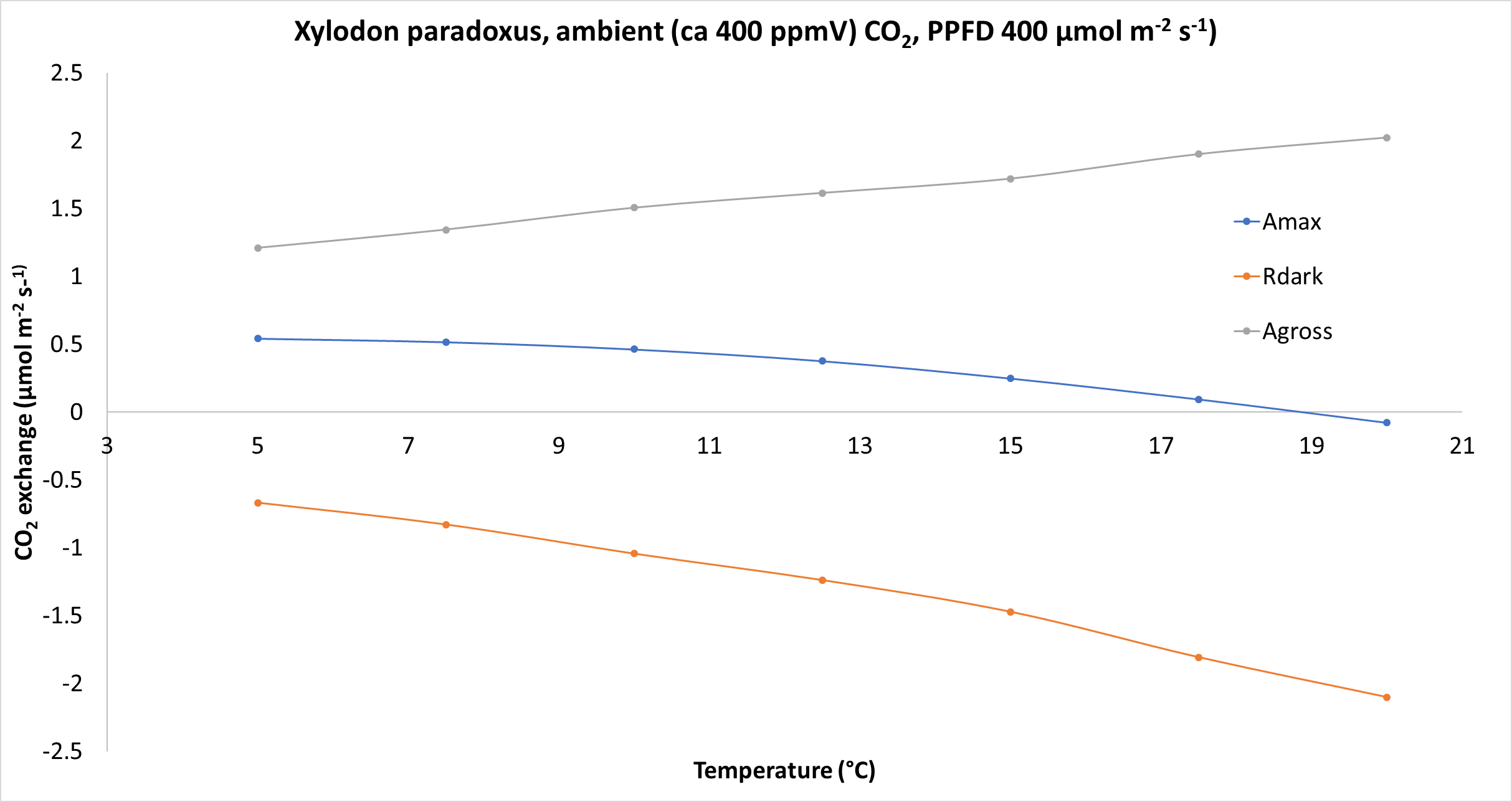


**Fig. S11 GC-IRMS analyses of trimethylsililated (TMS) ribitol (RT=388s) and mannitol (436s) in *Skvortzovia furfuracea-Coccomyxa* alcobiosis with abundant algae layer (green lines) and prevailing fungal crust (brown lines) after 18 h assimilation in ^13^CO_2_ atmosphere.** Principal polyols, algal ribitol and fungal mannitol, were always present in these associations (upper panel). ^13^C enrichment pattern differs strikingly between these two polyols (bottom panel). Whereas ribitol was *highly labelled, mannitol* ^13^C converges to natural level (1.07 ± 0.02 %, red arrow). About 100 mg of fresh weight or 2 to 6 cm^2^ of alcobioses were used in five replications. Black lines represent mixture of TMS ribitol and mannitol (25 µg ml^-1^ of each) of natural ^13^C signal. A ghost peaks before TMS-ribitol are non-fully sililated derivatises of ribitol, minor in abundance (upper panel) but having similar ^13^C pattern (bottom panel).


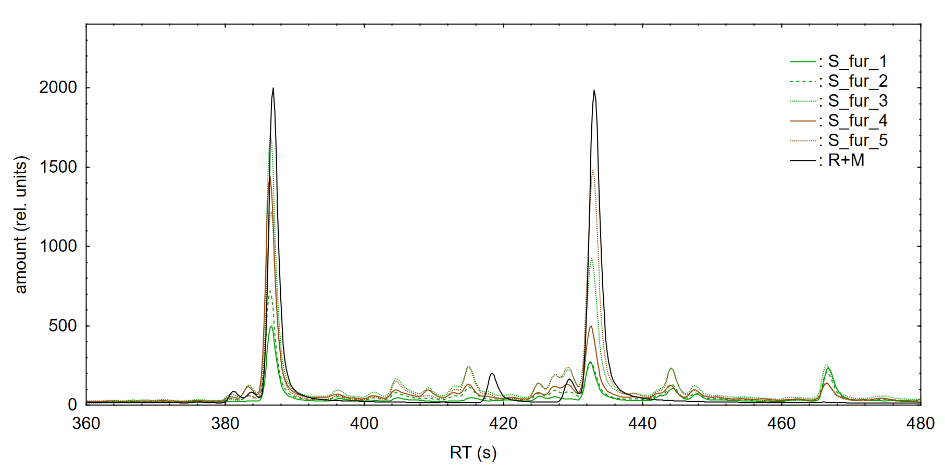

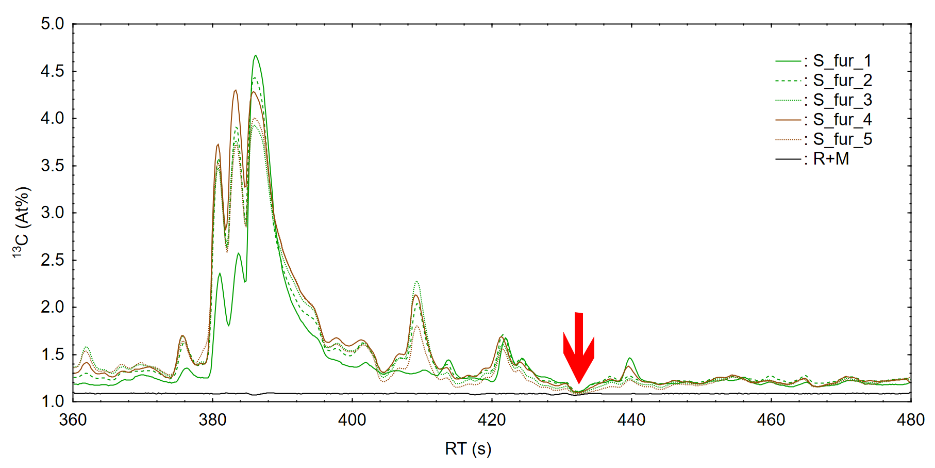


**Fig. S12 GC-IRMS analyses of trimethylsililated (TMS) ribitol (RT=925s), mannitol (1550s) and sorbitol (1575s) in *Botryobassidium botryosum-Coccomyxa* (red lines) and *Lyomyces sambuci-Desmococcus* (green lines) after 18 h assimilation in ^13^CO_2_ atmosphere.** *Botryobassidium botryosum-Coccomyxa* association has ribitol is a principal algal polyol, whereas *Lyomyces sambuci-Desmococcus* accumulates predominantly sorbitol (upper panel). ^13^C enrichment pattern differs strikingly between associations suggesting qualitative difference (bottom panel). Whereas in *Lyomyces sambuci-Desmococcus* sorbitol is highly labelled and substantial part of labelled carbon appears in fungal mannitol, in *Botryobassidium botryosum-Coccomyxa,* mannitol ^13^C converges to natural level (1.07 ± 0.02 %). Botryo_1 and Botryo_2, as well as, Lyo_sam1 and 2 are independent replications of alcobioses (about 100 mg of fresh weight or 2 to 6 cm^2^). Lyo_samR is neighbor algae cushion not covered by fungal crust but probably still with some fungal presence (see low mannitol content in upper panel). Lyo_samH, in contrast, is *Lyomyces* crust with low algal presence (see low sorbitol content). Despite this contrast, labelling pattern is almost the same. Black line represents mixture of TMS-ribitol, -mannitol and -sorbitol (25 µg ml^-1^ of each) of natural ^13^C signal.


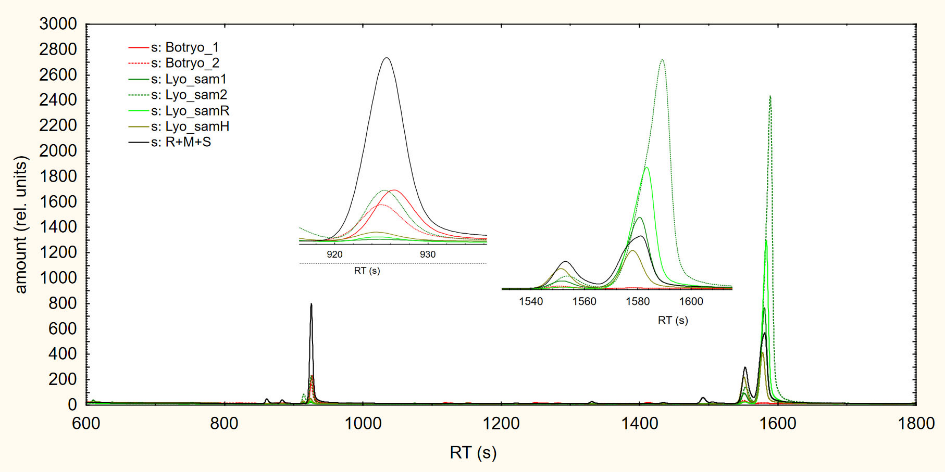

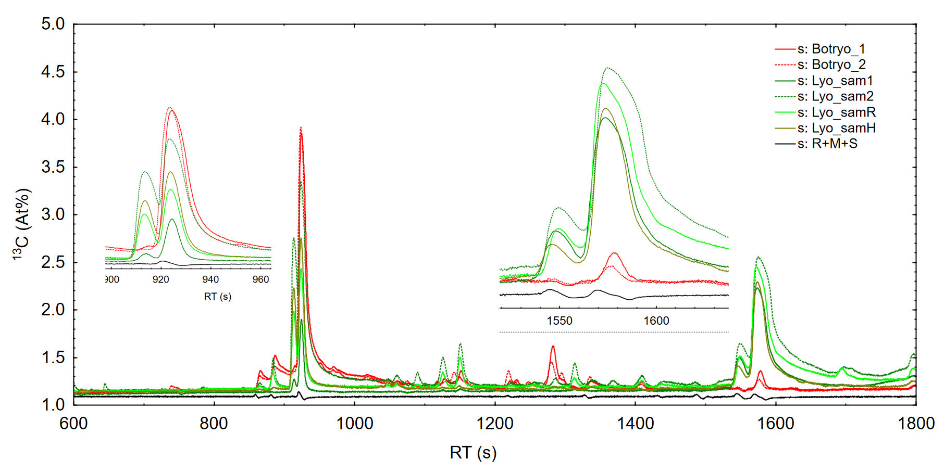


**Table S2** Loci used in this study

| Locus | Primer name | Sequence (5´–3´) | Annealing temp (C) | Source |
| --- | --- | --- | --- | --- |
| ITS | ITS1F | CTTGGTCATTTAGAGGAAGTAA | 55 | Gardes and Bruns 1993 |
| ITS | ITS4 | TCCTCCGCTTATTGATATGC | 55 | White et al. 1990 |
| rbcL | ORB1F | CCACAAACTGAAACAAAAGCA | 52 | Štenclová et al. 2017 |
| rbcL | ORB1R | CTGGAGCATTACCCCAAGG | 52 | Štenclová et al. 2017 |

Gardes M., Bruns T.D. (1993) ITS primers with enhanced specificity for basidiomycetes. Application for the identification of mycorrhizae and rust. Molecular Ecology 2: 113–118.

Štenclová L., Fučíková K., Kaštovský J., Pažoutová M. (2017) Molecular and morphological delimitation and generic classification of the family Oocystaceae (Trebouxiophyceae, Chlorophyta). Journal of Phycology 53: 1263–1282.

White T.J., Bruns T., Lee S., Taylor J.W. (1990) Amplification and direct sequencing of fungal ribosomal RNA genes for phylogenetics. In: Innis M.A., Gelfand D.H., Sninsky J.J., White T.J. eds. PCR protocols: A guide to the methods and applications. New York: Academic Press. 315–322.

Method S1:

**Metabolomics: Gas chromatography – mass spectrometry**

Homogenized and filtered MetOH extracts (see material and methods) were trimethylsilylated before GC analysis [1]. Samples in 2 mL chromatographic vials were dried out by gentle stream of nitrogen. Then, 100 µL of pyridine and 50 µL of BSTFA

(N,O-bis[trimethylsilyl]trifluoroacetamide) were added and heated the samples at 85 °C for one hour. Resulted trimethylsilyl (TMS-) derivatives were dissolved in hexane and analyzed compound-specific via gas chromatograph, GC (Trace 1310, Thermo, Bremen, Germany), injector at 300 °C. Injection was splitless for 1.5 min, then split 100 mL/min for next one min, and 5 mL/min (gas saver) for the rest of analysis. Eluting compounds were oxidized to CO_2_ via IsoLink II interphase (Thermo, Bremen, Germany) at 1000 °C and introduced to continuous-flow isotope ratio MS (Delta V Advantage, Thermo, Bremen, Germany). Two chromatographic columns and temperature programs were used.

Fast column LION LN-05 Sil-MS (30 m x 0.25 mm x 0.1 µm film thickness) with flow rate 1.5 mL/min of helium was used to resolve TMS-ribitol (C_20_H_52_O_5_Si_5_) and TMS-mannitol (C_24_H_62_O_6_Si_6_) in *Skvortzovia-Coccomyxa* system. Temperature program was: 50 °C during injection and for next two min, then 50 to 185 °C with slope 40 °C/min, 185 to 275 °C with slope 20 °C/min, 275 to 320 °C with slope 4 °C/min and hold 320 °C for the rest of time (total time ca 25 min).

To resolve TMS-sorbitol and TMS-mannitol (both C_24_H_62_O_6_Si_6_) in *Lyomyces-Desmococcus* system, we used column Restek Rxi-5Sil-MS (30m x 0.25 mm x 0.25 µm film thickness) and flow rate 1.5 mL/min of helium as a carrier. Oven temperature was 50°C during injection and for next two min, then increasing with slope 25°C/min to 150 °C, further 0.6°C/min to 170 °C (in this frame window both, mannitol and sorbitol elute), then 20 °C/min to 310 °C and isothermal at 310 °C for the rest of the analysis (*ca* 55 min in total).
